# Trends in mortality from non-natural causes in children and adolescents (0–19 years) in Europe from 2000 to 2018

**DOI:** 10.1186/s12889-023-17040-5

**Published:** 2023-11-10

**Authors:** Sara Tunesi, Stefano Tambuzzi, Adriano Decarli, Cristina Cattaneo, Antonio Giampiero Russo

**Affiliations:** 1 Epidemiology Unit, Agency for Health Protection of Milan, Via Conca del Naviglio, 45, 20123 Milano, (MI) Italy; 2 Bureau of Legal Medicine and Insurance, Department of Biomedical Science for Health, University of Milan, Milan, Italy

**Keywords:** Mortality, Non-natural causes, Children and adolescents, Mortality rates, Europe

## Abstract

**Background:**

Non-natural mortality in children and adolescents is a global public health problem that varies widely from country to country. Data on child and adolescent maltreatment are not readily available, and mortality due to violent causes is also underestimated.

**Methods:**

Injury-related mortality rates (overall and by specific causes) from 2000 to 2018 in selected European countries were analysed to observe mortality patterns in children and adolescents using data from the Eurostat database. Age-standardized mortality rates per 100,000 person-years were calculated for each country. *Joinpoint* regression analysis with a significance level of 0.05 and 95% confidence intervals was performed for mortality trends.

**Results:**

Children and adolescent mortality from non-natural causes decreased significantly in Europe from 10.48 around 2005 to 5.91 around 2015. The Eastern countries (Romania, Bulgaria, Poland, Slovakia, Czech Republic) had higher rates; while Spain, Denmark, Italy, and the United Kingdom had the lowest. Rates for European Country declined by 5.10% per year over the entire period. Larger downward trends were observed in Ireland, Spain and Portugal; smaller downward trends were observed for Eastern countries (Bulgaria, Czech Republic, Poland, Slovakia) and Finland. Among specific causes of death, the largest decreases were observed for accidental causes (-5.9%) and traffic accidents (-6.8%).

**Conclusions:**

Mortality among children and adolescents due to non-natural causes has decreased significantly over the past two decades. Accidental events and transport accidents recorded the greatest decline in mortality rates, although there are still some European countries where the number of deaths among children and adolescents from non-natural causes is high. Social, cultural, and health-related reasons may explain the observed differences between countries.

**Supplementary Information:**

The online version contains supplementary material available at 10.1186/s12889-023-17040-5.

## Key Messages

• The mortality of children and adolescents due to non-natural causes has decreased significantly in the last two decades;

• Substantial differences between countries were found in the trend of non-natural causes of death in children and adolescents;

• Accidental events and transport accidents showed the greatest decrease in mortality;

• In some European countries, there is still a high number of deaths in children and adolescents that are due to non-natural causes;

**Supplementary Information:**

The online version contains supplementary material available at 10.1186/s12889-023-17040-5.

## Introduction

The death of children and adolescents from non-natural causes is a tragic phenomenon that occurs in all countries of the world, although some countries are more affected than others. Although from 1990 to 2019, the under-20 years old mortality rate decreased in all countries, in 2019 alone, there were almost 1 million deaths in children and adolescents (5–14 years) worldwide [1], almost all of which were due to non-natural causes [2, 3]. From a medical perspective, natural death refers to deaths that occur solely as a result of disease or natural processes (e.g., old age or internal malfunctions of the body not directly caused by external forces). Natural deaths contrast with non-natural deaths, which include homicide, suicide, and accidents. In all of these cases, the causes are potentially preventable because an external and violent event occurs that leads to the person's death [4]. The 2030 Agenda for Sustainable Development [5] identified reducing under-5 years old mortality rates and preventing injuries, violence, harmful practices, and substance abuse among youth as key targets. In developed countries, deaths among children and adolescents have become rare thanks to declines in deaths from infectious diseases or cancer, as demonstrated by recent literature reports [6, 7]. However, injury-related causes remain the most common cause of death among children and adolescents [3, 8], and the number of children who are victims of neglect and/or non-accidental trauma is also often underreported [9–12]. In general, there are many reports of non-natural deaths in children and adolescents, but the crucial problem is that they are too rarely subjected to forensic autopsy [13, 14]. Particularly in the case of children, an autopsy is too often not performed because it is considered inappropriate, as it would violate the innocence of the young victims. Social, cultural, and/or religious factors may play a crucial role from country to country [15, 16]. However, this leads to a dangerous lack of forensic investigation. Especially in Italy, data on child and adolescent maltreatment are not readily available [17] and mortality due to violent causes is also underestimated [18, 19]. This fact was also confirmed by direct experience in the city of Milan. When evaluating the total number of children and adolescents recorded in the archives of the city of Milan who died from non-natural causes, we found that only about half of them were autopsied at the Institute of Forensic Medicine of Milan (one of the largest in Italy) [20, 21]. This represents an enormous loss of information, not only from a medico-legal point of view (i.e. signs of abuse or violence), but also from an epidemiological perspective and in terms of public health in its broadest sense. Moreover, such an attitude is not at all protective of a vulnerable group of people such as children and adolescents, who are at greater risk of becoming victims of direct and indirect violence [13, 22].

We therefore wondered whether such a critical attitude may be also found in other European countries and concluded that it is impossible to obtain reliable information on the number of autopsies (especially judicial ones) in each of these countries. Therefore, we decided to look at the issue of non-natural deaths in children and adolescents from a broader perspective. Knowing the most common causes of violent and traumatic deaths in children and adolescents and the places where they occurred can be an excellent starting point for considering this issue, at least from a European perspective. Even in high-income countries, it is important to monitor recent trends in non-natural child mortality so that policy makers and stakeholders can identify what actions can be taken to reduce it. This study is the first to examine mortality from non-natural (and thus potentially preventable) causes among children and adolescents in European countries. Depending on data availability, injury-related mortality rates from 2000 to 2018 in selected European countries were analysed to highlight recent mortality patterns in European children and adolescents using data from the Eurostat database. The aim is to give a systematic description of this phenomenon in the last two decades and to point out a reality that exists but has not yet been fully described in the literature. This information could be important to understand the vulnerabilities and allow the different involved countries to assess the causes of these events in order to develop prevention and awareness strategies to protect the youngest.

## Methods

### Population and outcome

The study population was the EU28 population and the population of European countries with at least 1,000,000 inhabitants aged 0–19 years in the years 2000–2018. As outcomes, deaths due to non-natural causes of morbidity and mortality recorded in the selected countries were considered.

### Data source

The number of deaths aged 0–19 years due to non-natural causes (i.e. external causes of morbidity and mortality according to ICD X V01-Y89) from 2000 to 2018 (or the last available calendar year) in different European countries was extracted from Eurostat databases [23]. Causes of deaths were recorded, for each country and calendar year according to the 10^th^ revision of the ICD (International Classification of Disesases) [24]. Mortality from external or non-natural causes (EXT ICDX codes: V01-Y89), accidental events (ACC ICDX codes: V01-X59, Y85, Y86), transport accidents (TA ICDX codes: V01-V99, Y85), intentional self-harm (SH ICDX codes:X60-X84, Y87.0), exposure to noxious harmful substances (POI ICDX codes: X40-X49), and assault (ASS ICDX codes: X85-Y09, Y87.1) were also extracted. Estimates of resident population based on the official census for the years studied were extracted from the same database.

### Statistical analysis

For each country, sex and calendar years, age-specific rates were estimated for 5 ages groups (< 1, 1–4, 5–9, 10–14, 15–19 years) and then age-standardized mortality rates (ASMR) per 100,000 person-years were calculated based on a world standard population [25].

*Joinpoint* regression analysis [26] of annual age-standardized mortality rates was performed for all the causes of death included in the study. Time points, referred to as *joinpoint*(s), were identified when a significant change in the linear slope (on the logarithmic scale) of the temporal trend occurred, using calendar years as regressor variable [27]. *Joinpoint* analysis started by fitting a minimum number of *joinpoint* (e.g. zero, a straight line) and then it tested [28] whether adding one or more *joinpoint* (up to three) to the model would have significantly improved the fit; if so, the *joinpoints* were added to the model and a linear regression was fitted between consecutive *joinpoints* using the least squares approach. Selection of the number of inflection points was performed through Monte Carlo permutation tests [27]. As a summary measure, the annual percent change (APC) for each identified linear segment and the weighted average APC (AAPC) over the entire study period (2000–2018) were estimated [29]. Analysis of rates was performed using R software [30]; the *Joinpoint* regression trend analysis and Annual Percentage Change (APC) were performed using *Joinpoint* Regression Program [26]. A significance level of α = 0.05 was applied to all analyses, and 95% confidence intervals were assumed; no corrections were made for missing data, so missing time point data were omitted [31].

## Results

Data from the 21 European countries selected according to the inclusion criteria were analysed. The age-standardized rates per 100,000 children aged 0 to 19 years and the number of deaths from non-natural causes, accidental events, transport accidents, intentional self-harm, and assault for selected countries and the EU28 in the first period around 2005 (2003–2007) and in the second period around 2015 (2013–2017) are shown in Table 1. For the period around 2015, Fig. 1 shows a ranking of reported ASMR from  the lowest to the highest, for boys and girls separately and together; Italy and the EU28 are highlighted.

**Table 1:** Number of death and age-standardized (word population) mortality rates at age 0–19 per 100,000 from External Causes, Accidents, Transport Accidents, Intentional self-harm, Poisoning and Assault in boys and girls and combinate around 2015 (2013–2017), for selected European Country and EU28

Country	Boys						Girls						Boys and girls					
	N. 2003–2007	ASM 2003–2007	CI 95%	N. 2013–2017	ASM 2013–2017	CI 95%	N. 2003–2007	ASM 2003–2007	CI 95%	N. 2013–2017	ASM 20013–2017	CI 95%	N. 2003–2007	ASM 2003–2007	CI 95%	N. 2013–2017	ASM 2013–2017	CI 95%
**External Causes (V01-Y89)**																		
Austria	822	16.73	15.60–17.92	484	10.28	9.38–11.24	316	6.92	6.17–7.73	181	4.20	3.61–4.86	1138	11.82	11.14–12.54	665	7.24	6.70–7.81
Belgium	960	15.17	14.22–16.16	586	8.97	8.26–9.73	438	7.35	6.68–8.07	269	4.33	3.83–4.88	1398	11.26	10.68–11.87	855	6.65	6.21–7.11
Bulgaria	918	21.08	19.70–22.52	503	14.99	13.71–16.36	456	11.77	10.69–12.93	193	6.08	5.25–7.00	1374	16.42	15.54–17.34	696	10.54	9.77–11.35
Switzerland	557	12.70	11.67–13.81	354	7.92	7.11–8.79	224	5.50	4.80–6.28	138	3.32	2.79–3.93	781	9.10	8.47–9.77	492	5.62	5.13–6.14
Czechia	1046	16.77	15.75–17.83	559	10.98	10.09–11.93	447	7.96	7.23–8.74	234	4.76	4.17–5.42	1493	12.36	11.74–13.02	793	7.87	7.33–8.44
Germany	5252	11.27	10.97–11.58	2791	6.78	6.53–7.04	2173	5.11	4.89–5.33	1212	3.20	3.02–3.39	7425	8.19	8.00–8.38	4003	4.99	4.84–5.15
Denmark	369	11.28	10.16–12.49	185	5.27	4.54–6.09	163	5.15	4.39–6.01	88	2.63	2.11–3.25	532	8.22	7.53–8.95	273	3.95	3.50–4.45
Greece	982	15.20	14.26–16.19	570	10.36	9.53–11.25	329	5.61	5.02–6.25	240	4.66	4.09–5.29	1311	10.41	9.85–10.99	810	7.51	7.00–8.05
Spain	3127	13.26	12.80–13.73	1081	4.66	4.39–4.95	1114	5.12	4.82–5.43	540	2.47	2.27–2.69	4241	9.19	8.91–9.47	1621	3.57	3.39–3.74
Finland	586	17.93	16.50–19.44	319	10.07	9.00–11.24	226	7.32	6.39–8.34	136	4.51	3.78–5.33	812	12.62	11.77–13.52	455	7.29	6.64–7.99
France	5691	13.62	13.26–13.97	3370	8.10	7.82–8.37	2148	5.45	5.22–5.68	1498	3.80	3.61–3.99	7839	9.53	9.32–9.75	4868	5.95	5.78–6.12
Hungary	919	15.17	14.20–16.19	456	8.70	7.92–9.54	392	7.02	6.34–7.76	179	3.72	3.19–4.31	1311	11.10	10.50–11.72	635	6.21	5.74–6.72
Ireland	476	15.61	14.24–17.08	211	6.66	5.79–7.62	168	5.82	4.97–6.77	72	2.38	1.86–2.99	644	10.72	9.90–11.58	283	4.52	4.01–5.08
Italy	2107	11.88	11.37–12.40	1802	6.07	5.80–6.36	726	4.38	4.07–4.72	644	2.35	2.17–2.54	2833	8.13	7.83–8.44	2446	4.21	4.05–4.38
Netherlands	904	8.87	8.31–9.47	609	6.02	5.55–6.52	419	4.32	3.92–4.75	291	3.03	2.69–3.40	1323	6.60	6.25–6.96	900	4.52	4.23–4.83
Poland	5220	18.81	18.30–19.34	2687	12.93	12.45–13.43	1976	7.99	7.63–8.36	1008	5.20	4.88–5.53	7196	13.40	13.08–13.72	3695	9.07	8.78–9.36
Portugal	954	15.76	14.77–16.79	370	6.83	6.15–7.57	367	6.53	5.88–7.24	134	2.64	2.21–3.13	1321	11.15	10.55–11.76	504	4.74	4.33–5.17
Romania	3951	28.83	27.92–29.76	1988	18.35	17.55–19.18	1957	15.79	15.08–16.52	909	9.04	8.46–9.65	5908	22.31	21.73–22.90	2897	13.7	13.20–14.21
Sweden	618	10.64	9.81–11.51	387	6.80	6.14–7.52	325	5.94	5.31–6.63	179	3.36	2.88–3.89	943	8.29	7.77–8.84	566	5.08	4.67–5.52
Slovakia	623	16.65	15.34–18.04	359	12.03	10.82–13.35	259	7.85	6.90–8.89	149	5.35	4.52–6.28	882	12.25	11.43–13.10	508	8.69	7.95–9.48
UK	4416	11.21	10.88–11.55	2325	5.85	5.61–6.09	1777	4.79	4.57–5.02	998	2.65	2.49–2.82	6193	8.00	7.80–8.20	3323	4.25	4.11–4.40
**EU28**	**44,241**	**14.62**	**14.49–14.76**	**22,765**	**8.15**	**8.05–8.26**	**17,714**	**6.34**	**6.25–6.44**	**9574**	**3.66**	**3.59–3.74**	**61,955**	**10.48**	**10.4–10.57**	**32,339**	**5.91**	**5.84–5.97**
**Accidental Causes (V01-X59. Y85. Y86)**																		
Austria	562	11.49	10.56–12.49	272	5.82	5.14–6.55	216	4.73	4.12–5.41	91	2.12	1.71–2.61	778	8.11	7.55–8.70	363	3.97	3.57–4.40
Belgium	680	10.77	9.98–11.62	346	5.30	4.75–5.88	282	4.75	4.22–5.34	156	2.51	2.13–2.94	962	7.76	7.28–8.27	502	3.90	3.57–4.26
Bulgaria	764	17.8	16.53–19.14	402	11.97	10.83–13.2	376	9.97	8.96–11.05	150	4.73	4.00–5.55	1140	13.89	13.07–14.74	552	8.35	7.67–9.08
Switzerland	389	8.93	8.06–9.87	211	4.76	4.14–5.45	130	3.23	2.70–3.84	80	1.95	1.54–2.43	519	6.08	5.57–6.63	291	3.36	2.98–3.76
Czechia	763	12.35	11.48–13.27	372	7.24	6.52–8.02	343	6.21	5.56–6.91	140	2.83	2.38–3.34	1106	9.28	8.73–9.85	512	5.03	4.61–5.49
Germany	3861	8.33	8.06–8.59	1763	4.32	4.12–4.52	1630	3.84	3.65–4.03	678	1.81	1.68–1.95	5491	6.08	5.92–6.25	2441	3.06	2.94–3.19
Denmark	286	8.74	7.76–9.82	109	3.13	2.57–3.77	130	4.09	3.42–4.86	51	1.53	1.14–2.02	416	6.42	5.82–7.07	160	2.33	1.98–2.72
Greece	927	14.35	13.44–15.31	502	9.12	8.34–9.95	307	5.24	4.67–5.87	215	4.18	3.64–4.78	1234	9.8	9.25–10.36	717	6.65	6.17–7.15
Spain	2797	11.88	11.45–12.33	779	3.35	3.12–3.59	959	4.42	4.14–4.71	372	1.70	1.53–1.88	3756	8.15	7.89–8.42	1151	2.53	2.38–2.68
Finland	373	11.50	10.36–12.73	189	5.99	5.16–6.90	151	4.95	4.19–5.80	70	2.34	1.82–2.96	524	8.23	7.53–8.96	259	4.16	3.67–4.70
France	4523	10.84	10.52–11.16	2517	6.06	5.82–6.30	1671	4.26	4.06–4.47	1075	2.73	2.57–2.90	6194	7.55	7.36–7.74	3592	4.39	4.25–4.54
Hungary	670	11.19	10.36–12.08	298	5.74	5.11–6.43	289	5.17	4.59–5.81	115	2.40	1.98–2.89	959	8.18	7.67–8.72	413	4.07	3.69–4.49
Ireland	301	9.92	8.83–11.11	122	3.81	3.16–4.55	111	3.87	3.18–4.66	33	1.06	0.73–1.49	412	6.89	6.24–7.59	155	2.43	2.06–2.85
Italy	1871	10.55	10.08–11.04	1435	4.84	4.60–5.10	628	3.79	3.50–4.10	491	1.80	1.65–1.97	2499	7.17	6.89–7.46	1926	3.32	3.18–3.47
Netherlands	631	6.19	5.72–6.70	379	3.78	3.41–4.19	295	3.04	2.70–3.40	134	1.43	1.19–1.69	926	4.62	4.32–4.92	513	2.61	2.38–2.84
Poland	3471	12.88	12.45–13.33	1710	8.27	7.88–8.67	1517	6.22	5.90–6.55	706	3.66	3.39–3.94	4988	9.55	9.28–9.83	2416	5.97	5.73–6.21
Portugal	748	12.36	11.49–13.28	258	4.81	4.24–5.44	281	5.02	4.45–5.64	88	1.75	1.40–2.16	1029	8.69	8.16–9.24	346	3.28	2.94–3.64
Romania	3306	24.55	23.7–25.42	1633	15.15	14.43–15.91	1725	14.11	13.44–14.81	756	7.56	7.03–8.12	5031	19.33	18.79–19.88	2389	11.36	10.9–11.82
Sweden	427	7.46	6.76–8.20	192	3.37	2.91–3.88	198	3.68	3.19–4.24	65	1.21	0.93–1.54	625	5.57	5.14–6.03	257	2.29	2.02–2.59
Slovakia	475	12.84	11.68–14.07	256	8.64	7.61–9.77	206	6.31	5.46–7.26	106	3.82	3.12–4.62	681	9.57	8.85–10.34	362	6.23	5.60–6.91
UK	3087	7.85	7.57–8.13	1477	3.72	3.53–3.92	1169	3.16	2.98–3.35	628	1.67	1.54–1.81	4256	5.50	5.34–5.67	2105	2.70	2.58–2.81
**EU28**	**33,948**	**11.29**	**11.17–11.41**	**15,740**	**5.66**	**5.57–5.75**	**13,720**	**4.95**	**4.87–5.03**	**6392**	**2.46**	**2.40–2.52**	**47,668**	**8.12**	**8.05–8.19**	**22,132**	**4.06**	**4.00–4.11**
**Transport accidents (v01-V99. Y85)**																		
Austria	383	7.69	6.93–8.50	157	3.29	2.80–3.85	161	3.44	2.92–4.01	61	1.41	1.08–1.81	544	5.56	5.10–6.05	218	2.35	2.05–2.69
Belgium	470	7.33	6.68–8.03	208	3.16	2.74–3.62	174	2.87	2.46–3.34	90	1.44	1.16–1.77	644	5.10	4.72–5.51	298	2.30	2.04–2.57
Bulgaria	319	6.84	6.10–7.66	195	5.80	5.02–6.68	186	4.55	3.91–5.27	68	2.15	1.67–2.72	505	5.70	5.20–6.23	263	3.97	3.51–4.49
Switzerland	232	5.25	4.60–5.97	92	2.05	1.65–2.52	65	1.59	1.23–2.03	42	1.02	0.73–1.38	297	3.42	3.04–3.84	134	1.54	1.29–1.82
Czechia	473	7.37	6.71–8.07	221	4.37	3.81–4.99	209	3.6	3.12–4.13	80	1.63	1.29–2.03	682	5.49	5.08–5.92	301	3.00	2.67–3.36
Germany	2858	5.93	5.72–6.15	1128	2.69	2.53–2.85	1164	2.61	2.46–2.76	440	1.15	1.04–1.26	4022	4.27	4.14–4.41	1568	1.92	1.82–2.01
Denmark	197	6.06	5.24–6.96	67	1.86	1.44–2.37	86	2.72	2.17–3.36	33	0.98	0.68–1.38	283	4.39	3.89–4.93	100	1.42	1.16–1.73
Greece	692	10.57	9.79–11.39	388	7.04	6.35–7.77	231	3.90	3.41–4.44	169	3.27	2.80–3.80	923	7.23	6.77–7.72	557	5.15	4.73–5.60
Spain	1921	8.06	7.71–8.43	370	1.60	1.44–1.77	656	2.98	2.76–3.22	196	0.90	0.77–1.03	2577	5.52	5.31–5.74	566	1.25	1.15–1.35
Finland	221	6.74	5.88–7.69	120	3.79	3.14–4.53	86	2.77	2.21–3.42	43	1.43	1.04–1.93	307	4.75	4.23–5.32	163	2.61	2.23–3.05
France	2883	6.81	6.56–7.06	1264	3.02	2.85–3.19	959	2.40	2.25–2.56	509	1.28	1.17–1.39	3842	4.60	4.46–4.75	1773	2.15	2.05–2.25
Hungary	391	6.33	5.71–6.99	133	2.50	2.09–2.96	180	3.11	2.67–3.61	61	1.23	0.94–1.58	571	4.72	4.34–5.13	194	1.86	1.61–2.15
Ireland	185	6.06	5.22–7.00	57	1.79	1.35–2.31	72	2.48	1.94–3.13	18	0.58	0.34–0.92	257	4.27	3.76–4.83	75	1.18	0.93–1.48
Italy	1392	7.81	7.40–8.23	909	3.05	2.85–3.25	463	2.77	2.52–3.03	301	1.09	0.97–1.22	1855	5.29	5.05–5.53	1210	2.07	1.95–2.19
Netherlands	415	4.06	3.68–4.47	224	2.18	1.90–2.49	209	2.14	1.86–2.45	81	0.83	0.66–1.03	624	3.10	2.86–3.35	305	1.51	1.34–1.68
Poland	2088	7.48	7.15–7.81	1068	5.13	4.83–5.45	1012	3.94	3.70–4.20	484	2.48	2.27–2.72	3100	5.71	5.51–5.92	1552	3.81	3.62–4.00
Portugal	526	8.61	7.89–9.38	150	2.70	2.28–3.17	199	3.49	3.02–4.02	46	0.88	0.64–1.17	725	6.05	5.62–6.51	196	1.79	1.55–2.06
Romania	1097	7.67	7.21–8.14	689	6.27	5.81–6.76	641	4.77	4.40–5.16	356	3.45	3.10–3.83	1738	6.22	5.92–6.52	1045	4.86	4.57–5.16
Sweden	230	3.89	3.41–4.43	86	1.52	1.21–1.87	91	1.63	1.31–2.00	34	0.64	0.44–0.89	321	2.76	2.47–3.08	120	1.08	0.89–1.29
Slovakia	258	6.65	5.85–7.52	125	4.16	3.46–4.95	130	3.71	3.08–4.42	55	1.94	1.46–2.52	388	5.18	4.67–5.73	180	3.05	2.62–3.53
UK	2175	5.45	5.23–5.69	529	1.33	1.22–1.45	770	2.04	1.90–2.19	214	0.57	0.49–0.65	2945	3.75	3.61–3.88	743	0.95	0.88–1.02
**EU28**	**21,384**	**6.92**	**6.83–7.02**	**8472**	**3.01**	**2.95–3.08**	**8449**	**2.94**	**2.88–3.01**	**3492**	**1.32**	**1.28–1.37**	**29,833**	**4.93**	**4.88–4.99**	**11,964**	**2.17**	**2.13–2.21**
**Intentional self-harm (X60-X84 Y87.0)**																		
Austria	195	3.82	3.31–4.40	125	2.56	2.13–3.06	53	1.09	0.82–1.43	48	1.06	0.78–1.41	248	2.46	2.16–2.78	173	1.81	1.55–2.11
Belgium	190	2.93	2.53–3.37	160	2.43	2.07–2.84	80	1.29	1.02–1.60	63	1.00	0.77–1.28	270	2.11	1.86–2.37	223	1.72	1.50–1.96
Bulgaria	87	1.72	1.37–2.12	50	1.50	1.11–1.97	52	1.11	0.83–1.46	17	0.54	0.31–0.86	139	1.41	1.19–1.67	67	1.02	0.79–1.29
Switzerland	126	2.77	2.31–3.30	122	2.67	2.21–3.18	62	1.44	1.10–1.84	47	1.10	0.81–1.46	188	2.10	1.81–2.43	169	1.88	1.61–2.19
Czechia	174	2.59	2.22–3.00	145	2.92	2.47–3.44	48	0.76	0.56–1.00	55	1.17	0.88–1.52	222	1.67	1.46–1.91	200	2.05	1.77–2.35
Germany	913	1.85	1.73–1.98	720	1.68	1.56–1.80	294	0.63	0.56–0.71	337	0.85	0.76–0.94	1207	1.24	1.17–1.31	1057	1.26	1.19–1.34
Denmark	46	1.41	1.04–1.89	51	1.39	1.03–1.82	14	0.45	0.25–0.76	27	0.78	0.51–1.14	60	0.93	0.71–1.2	78	1.08	0.86–1.35
Greece	31	0.47	0.32–0.66	30	0.53	0.36–0.76	16	0.26	0.15–0.42	9	0	0–0	47	0.36	0.27–0.48	39	0.35	0.25–0.48
Spain	219	0.90	0.79–1.03	218	0.95	0.83–1.08	84	0.37	0.29–0.46	116	0.53	0.44–0.64	303	0.64	0.57–0.71	334	0.74	0.66–0.82
Finland	165	4.91	4.19–5.72	106	3.32	2.72–4.01	57	1.77	1.34–2.30	45	1.47	1.08–1.97	222	3.34	2.92–3.81	151	2.40	2.03–2.81
France	803	1.89	1.76–2.02	556	1.32	1.21–1.43	297	0.72	0.64–0.81	282	0.70	0.62–0.79	1100	1.30	1.23–1.38	838	1.01	0.94–1.08
Hungary	183	2.80	2.41–3.23	104	1.90	1.55–2.30	49	0.78	0.57–1.03	30	0.58	0.39–0.83	232	1.79	1.56–2.03	134	1.24	1.04–1.47
Ireland	145	4.71	3.98–5.55	79	2.54	2.01–3.17	37	1.26	0.89–1.74	36	1.22	0.85–1.69	182	2.99	2.57–3.46	115	1.88	1.55–2.26
Italy	169	0.94	0.80–1.09	298	0.99	0.88–1.11	48	0.28	0.21–0.37	113	0.40	0.33–0.48	217	0.61	0.53–0.7	411	0.69	0.63–0.77
Netherlands	154	1.50	1.27–1.76	181	1.72	1.48–1.99	62	0.63	0.49–0.81	116	1.16	0.96–1.39	216	1.07	0.93–1.22	297	1.44	1.28–1.61
Poland	1259	4.10	3.88–4.34	712	3.37	3.13–3.63	233	0.81	0.71–0.92	187	0.94	0.81–1.09	1492	2.46	2.33–2.59	899	2.16	2.02–2.3
Portugal	47	0.75	0.55–1.00	46	0.80	0.58–1.06	19	0.32	0.19–0.50	20	0.37	0.22–0.57	66	0.54	0.42–0.68	66	0.58	0.45–0.74
Romania	416	2.60	2.36–2.87	266	2.36	2.08–2.66	126	0.82	0.68–0.97	73	0.69	0.54–0.86	542	1.71	1.57–1.86	339	1.52	1.36–1.69
Sweden	142	2.34	1.98–2.76	128	2.26	1.89–2.69	93	1.62	1.30–1.98	85	1.61	1.29–2.00	235	1.98	1.74–2.25	213	1.94	1.69–2.22
Slovakia	86	2.02	1.62–2.50	48	1.56	1.15–2.07	15	0.36	0.20–0.60	13	0.46	0.24–0.78	101	1.19	0.97–1.45	61	1.01	0.77–1.30
UK	456	1.13	1.03–1.24	561	1.40	1.29–1.52	173	0.45	0.38–0.52	222	0.59	0.51–0.67	629	0.79	0.73–0.85	783	0.99	0.93–1.07
**EU28**	**6480**	**2.04**	**1.99–2.09**	**4873**	**1.71**	**1.67–1.76**	**2017**	**0.67**	**0.64–0.70**	**1980**	**0.74**	**0.71–0.77**	**8497**	**1.36**	**1.33–1.39**	**6853**	**1.23**	**1.20–1.26**
**Poisoning (X40-X49)**																		
Austria	7	0	0–0	10	0.22	0.10–0.40	4	0	0–0	4	0	0–0	11	0.11	0.06–0.20	14	0.15	0.08–0.25
Belgium	28	0.44	0.29–0.64	13	0.20	0.11–0.34	20	0.33	0.20–0.50	6	0	0–0	48	0.38	0.28–0.51	19	0.15	0.09–0.23
Bulgaria	26	0.60	0.39–0.89	3	0	0–0	17	0.44	0.25–0.70	8	0	0–0	43	0.52	0.37–0.70	11	0.17	0.08–0.30
Switzerland	8	0	0–0	18	0.39	0.23–0.62	12	0.28	0.14–0.48	3	0	0–0	20	0.23	0.14–0.35	21	0.23	0.14–0.35
Czechia	31	0.49	0.33–0.69	16	0.32	0.18–0.52	20	0.35	0.21–0.54	13	0.27	0.15–0.47	51	0.42	0.31–0.55	29	0.30	0.20–0.43
Germany	69	0.15	0.11–0.19	51	0.12	0.09–0.16	37	0.09	0.06–0.12	25	0.07	0.04–0.10	106	0.12	0.10–0.14	76	0.09	0.07–0.12
Denmark	19	0.59	0.35–0.91	8	0	0–0	5	0	0–0	8	0	0–0	24	0.38	0.24–0.56	16	0.23	0.13–0.37
Greece	52	0.77	0.57–1.01	17	0.30	0.18–0.49	12	0.19	0.10–0.34	6	0	0–0	64	0.48	0.37–0.62	23	0.21	0.13–0.31
Spain	52	0.22	0.16–0.28	26	0.11	0.07–0.16	29	0.14	0.09–0.19	15	0.07	0.04–0.11	81	0.18	0.14–0.22	41	0.09	0.06–0.12
Finland	47	1.41	1.03–1.87	25	0.78	0.50–1.15	6	0	0–0	9	0	0–0	53	0.80	0.60–1.04	34	0.53	0.37–0.75
France	90	0.22	0.17–0.27	61	0.15	0.11–0.19	70	0.18	0.14–0.22	26	0.07	0.04–0.10	160	0.20	0.17–0.23	87	0.11	0.08–0.13
Hungary	35	0.57	0.40–0.80	20	0.38	0.23–0.59	22	0.38	0.24–0.57	8	0	0–0	57	0.47	0.36–0.62	28	0.27	0.18–0.39
Ireland	26	0.85	0.55–1.24	17	0.55	0.32–0.88	9	0	0–0	4	0	0–0	35	0.58	0.40–0.81	21	0.34	0.21–0.52
Italy	18	0.10	0.06–0.16	25	0.08	0.05–0.12	11	0.07	0.03–0.12	12	0.04	0.02–0.07	29	0.08	0.06–0.12	37	0.06	0.04–0.09
Netherlands	11	0.11	0.05–0.19	11	0.10	0.05–0.19	8	0	0–0	4	0	0–0	19	0.10	0.06–0.15	15	0.07	0.04–0.12
Poland	126	0.47	0.39–0.56	60	0.29	0.22–0.37	89	0.35	0.28–0.43	40	0.21	0.15–0.29	215	0.41	0.36–0.47	100	0.25	0.20–0.30
Portugal	12	0.21	0.11–0.37	2	0	0–0	8	0	0–0	3	0	0–0	20	0.18	0.11–0.27	5	0	0–0
Romania	270	2.21	1.95–2.5	83	0.79	0.63–0.98	225	1.89	1.65–2.16	71	0.72	0.56–0.91	495	2.05	1.87–2.24	154	0.75	0.64–0.88
Sweden	20	0.34	0.21–0.52	39	0.69	0.49–0.94	10	0.17	0.08–0.32	5	0	0–0	30	0.26	0.17–0.37	44	0.39	0.28–0.52
Slovakia	9	0	0–0	7	0	0–0	4	0	0–0	2	0	0–0	13	0.16	0.08–0.27	9	0	0–0
UK	131	0.33	0.27–0.39	181	0.45	0.39–0.52	82	0.21	0.17–0.26	88	0.23	0.19–0.29	213	0.27	0.23–0.31	269	0.34	0.30–0.39
**EU28**	**1218**	**0.41**	**0.39–0.43**	**707**	**0.25**	**0.23–0.27**	**749**	**0.27**	**0.25–0.29**	**376**	**0.14**	**0.13–0.16**	**1967**	**0.34**	**0.32–0.35**	**1083**	**0.20**	**0.19–0.21**
**Assault (X85-Y09 Y87.1)**																		
Austria	32	0.73	0.50–1.03	11	0.26	0.13–0.47	24	0.58	0.37–0.86	15	0.38	0.21–0.62	56	0.65	0.49–0.85	26	0.32	0.21–0.47
Belgium	62	1.01	0.78–1.30	33	0.51	0.35–0.72	56	0.97	0.73–1.25	26	0.42	0.28–0.62	118	0.99	0.82–1.18	59	0.47	0.36–0.60
Bulgaria	32	0.73	0.50–1.04	13	0.39	0.21–0.67	15	0.37	0.21–0.62	14	0.44	0.24–0.73	47	0.55	0.40–0.74	27	0.41	0.27–0.60
Switzerland	22	0.55	0.34–0.83	15	0.35	0.20–0.58	26	0.69	0.45–1.01	9	0	0–0	48	0.62	0.46–0.82	24	0.29	0.19–0.43
Czechia	21	0.39	0.24–0.60	14	0.27	0.15–0.45	14	0.25	0.13–0.42	20	0.38	0.23–0.59	35	0.32	0.22–0.45	34	0.32	0.22–0.45
Germany	194	0.48	0.42–0.56	144	0.38	0.32–0.45	149	0.39	0.33–0.46	114	0.31	0.26–0.38	343	0.44	0.39–0.49	258	0.35	0.31–0.39
Denmark	24	0.73	0.47–1.08	22	0.66	0.41–1.00	14	0.44	0.24–0.75	6	0	0–0	38	0.59	0.41–0.80	28	0.43	0.28–0.62
Greece	24	0.39	0.25–0.58	25	0.47	0.31–0.70	6	0	0–0	11	0.21	0.11–0.38	30	0.25	0.17–0.35	36	0.34	0.24–0.47
Spain	89	0.38	0.30–0.47	66	0.29	0.22–0.36	50	0.23	0.17–0.30	48	0.22	0.16–0.29	139	0.30	0.26–0.36	114	0.25	0.21–0.30
Finland	28	0.90	0.60–1.30	13	0.41	0.22–0.71	14	0.46	0.25–0.78	13	0.43	0.23–0.74	42	0.68	0.49–0.92	26	0.42	0.28–0.62
France	209	0.52	0.45–0.59	143	0.35	0.30–0.41	126	0.33	0.27–0.39	90	0.23	0.19–0.29	335	0.42	0.38–0.47	233	0.29	0.26–0.33
Hungary	40	0.77	0.55–1.05	23	0.48	0.30–0.72	51	1.01	0.75–1.34	25	0.55	0.35–0.81	91	0.89	0.72–1.10	48	0.51	0.38–0.68
Ireland	14	0.46	0.25–0.77	6	0	0–0	9	0	0–0	1	0	0–0	23	0.38	0.24–0.58	7	0	0–0
Italy	57	0.33	0.25–0.43	60	0.21	0.16–0.27	39	0.24	0.17–0.33	37	0.14	0.10–0.19	96	0.29	0.23–0.35	97	0.17	0.14–0.210
Netherlands	80	0.79	0.63–0.99	38	0.40	0.28–0.54	44	0.46	0.34–0.62	30	0.32	0.22–0.46	124	0.63	0.52–0.75	68	0.36	0.28–0.45
Poland	104	0.42	0.34–0.52	44	0.22	0.16–0.30	71	0.33	0.26–0.42	27	0.14	0.09–0.21	175	0.38	0.32–0.44	71	0.18	0.14–0.23
Portugal	41	0.68	0.48–0.92	20	0.37	0.22–0.57	17	0.31	0.18–0.49	7	0	0–0	58	0.49	0.37–0.63	27	0.26	0.17–0.38
Romania	159	1.13	0.96–1.33	48	0.45	0.33–0.60	64	0.52	0.39–0.66	47	0.47	0.35–0.63	223	0.82	0.72–0.94	95	0.46	0.37–0.56
Sweden	18	0.31	0.19–0.50	40	0.70	0.50–0.96	22	0.41	0.26–0.63	16	0.30	0.17–0.48	40	0.36	0.26–0.50	56	0.50	0.38–0.65
Slovakia	27	0.81	0.53–1.19	4	0	0–0	14	0.44	0.24–0.75	10	0.36	0.17–0.66	41	0.62	0.44–0.85	14	0.25	0.14–0.42
UK	127	0.33	0.27–0.39	26	0.07	0.04–0.10	64	0.18	0.14–0.23	23	0.06	0.04–0.09	191	0.25	0.22–0.29	49	0.06	0.05–0.08
**EU28**	**1531**	**0.53**	**0.50–0.56**	**835**	**0.31**	**0.29–0.33**	**952**	**0.36**	**0.33–0.38**	**610**	**0.24**	**0.22–0.26**	**2483**	**0.44**	**0.43–0.46**	**1445**	**0.27**	**0.26–0.29**

**Fig. 1:**
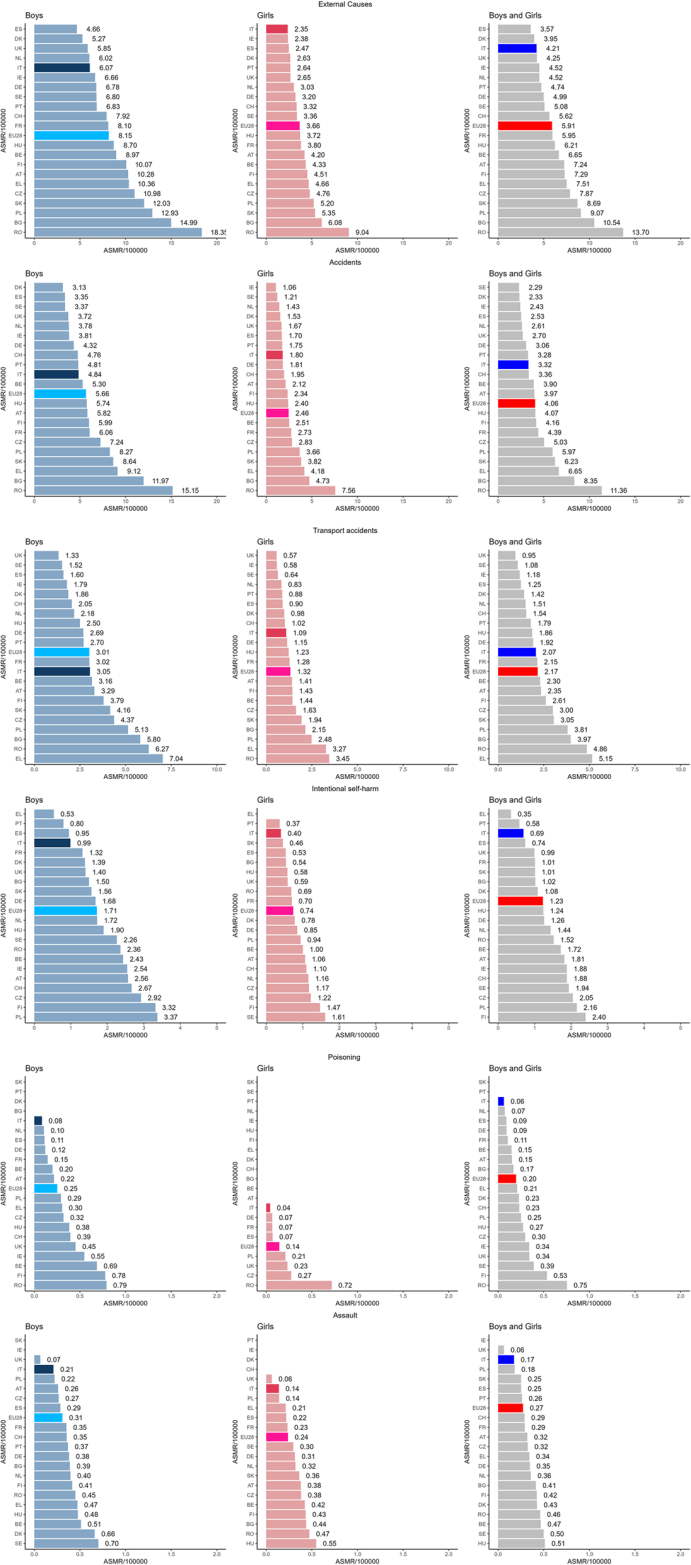
Bar plots of age-standardized (World population) mortality rates (ASMRs) per 100,000 (aged 0–19 years) from External Causes, Accidents, Transport Accidents, Intentional self-harm, Poisoning and Assault in boys and girls and combinate, ordered from the lowest to the highest around 2015 (2013–2017), for selected European Country and EU28; Italy and EU28 are highlighted

In the EU28, mortality from external causes decreased from 10.48 around 2005 to 5.91 around 2015. The individual analysed causes also decreased over the periods: mortality rates for accidental events decreased from 8.12 (95% CI 8.05–8.19) to 4.06 (95% CI 4.06–4.11); transport accidents rates decreased from 4.93 (95% CI 4.88–4.99) to 2.17 (95% CI 2.13—2.21); intentional self-harms rates decreased slightly from 1.36 (95% CI 1.22–1.39) to 1.23 (95% CI 1.20–1.26); poisoning rates decreased from 0.34 (95% CI 0.32–0.35) to 0.20 (95% CI 0.19–0.21); assault rates decreased from 0.44 (95% CI 0.43–0.46) to 0.27 (95% CI 0.26–0.29). In general, rates were higher for boys than for girls, and a comparison between the sexes showed a similar country rank for the same cause.

For non-natural causes the eastern countries (Romania, Bulgaria, Poland, Slovakia, Czech Republic) had higher rates; Switzerland, France and Hungary had rates similar to the EU28. Spain, Denmark, Italy, and the United Kingdom had the lowest rates.

For accidental events, Romania, Bulgaria, Greece, and Slovakia had higher mortality rates; Austria, Finland, Hungary, and Belgium had rates similar to the EU28; Sweden, Denmark, Ireland and Spain had the lowest rates.

Greece, Romania, and Bulgaria had higher transport accident rates. Italy, France, Belgium and Austria had rates similar to the EU28; the United Kingdom, Sweden, and Ireland had the lowest rates.

For intentional self-inflicted injuries, Finland, Poland and, the Czech Republic had the highest rates. Greece, Portugal, and Italy had the lowest rates for boys and girls combined. In all countries, rates were lower for girls than for boys; however, not all countries showed a decrease between 2005 and 2015, especially for girls.

Poisoning and assault rates were very close between countries, and in some cases the number of reported observations was close to zero or the time series were incomplete, so rates for these countries (i.e. Denmark, Netherlands, Slovakia, Portugal and Switzerland for poisoning; Ireland and Greece for assault) were not estimated. The highest rates for poisoning were recorded in Romania and Finland, and the lowest in Italy, Netherlands, Spain and Germany. The highest rates for assault were recorded in Hungary, Sweden, Belgium, and Romania, and the lowest in the United Kingdom, Italy and Poland.

**Fig. 2:**
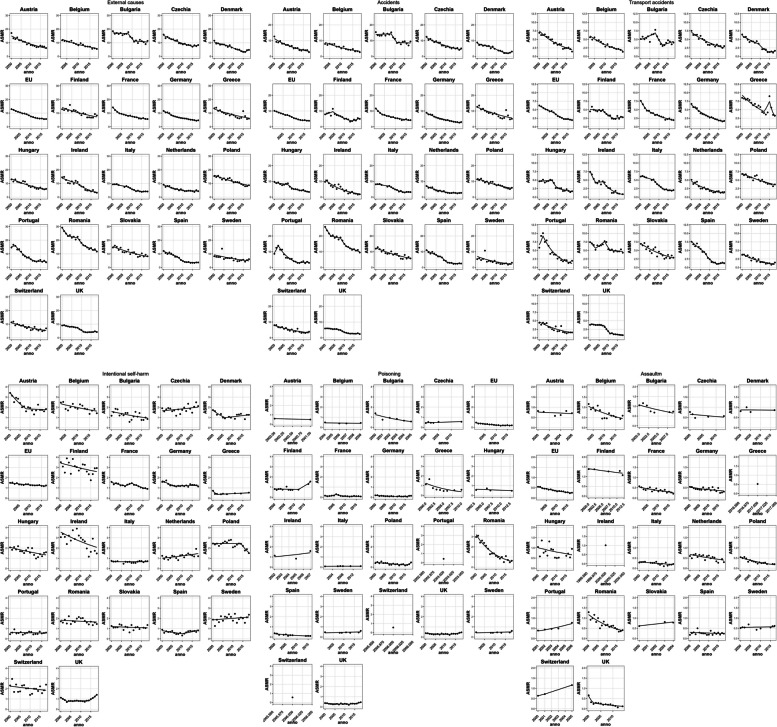
Age-standardized (World population) mortality rates (ASMRs) per 100,000 (aged 0–19 years) from External Causes, Accidents, Transport Accidents, Intentional self-harm, Poisoning and Assault over the 2000–2018 according to data availability. For Poisoning, Country without available data (Denmark, Netherlands and Slovakia) were not displayed

Figure 2 shows the trend in age-standardized mortality rates for the selected causes from 2000 to 2018 for the 21 European countries plus EU28, and Table 2 presents the estimates of APCs and AAPCs resulting from the *joinpoint* analysis. To avoid instability of APCs and AAPCs because of the small number of events in each country, analyses were performed for boys and girls and for combinations; the separate analyses are presented in the Supplementary Materials.

**Table 2:** *Joinpoint* analysis for External causes Accidents, Transport Accidents, Intentional self-harm, Poisoning and Assault from 2000 to 2018 (according to data availability) by country

Country	Trend 1	APC 1	Trend 2	APC 2	Trend 3	APC 3	Trend 4	APC 4	AAPC
**External causes**									
Austria	2000–2018	-5.0 (-5.7, -4.2)*							-5.0 (-5.7, -4.2)*
Belgium	2003–2018	-4.9 (-5.8, -4.0)*							-4.9 (-5.8, -4.0)*
Bulgaria	2000–2008	-0.5 (-2.5, 1.5)	2008–2011	-11.9 (-29.2, 9.5)	2011–2018	-1.0 (-4.4, 2.5)			-2.7 (-6.1, 0.8)
Switzerland	2000–2018	-4.0 (-5.1, -3.0)*							-4.0 (-5.1, -3.0)*
Czechia	2000–2015	-4.8 (-5.4, -4.1)*	2015–2018	3.7 (-6.3, 14.8)					-3.4 (-5.0, -1.8)*
Germany	2000–2007	-7.0 (-8.0, -5.9)*	2007–2018	-4.0 (-4.8, -3.3)*					-5.2 (-5.7, -4.6)*
Denmark	2000–2008	-4.3 (-6.3, -2.3)*	2008–2016	-9.6 (-12.8, -6.2)*	2016–2018	23.5 (-4.4, 59.5)			-4.0 (-6.8, -1.1)*
Greece	2000–2018	-4.0 (-5.3, -2.6)*							-4.0 (-5.3, -2.6)*
Spain	2000–2007	-5.4 (-7.6, -3.1)*	2007–2011	-16.4 (-25.6, -6.0)*	2011–2018	-1.9 (-5.7, 2.0)			-6.7 (-9.2, -4.0)*
Finland	2000–2018	-3.6 (-4.9, -2.4)*							-3.6 (-4.9, -2.4)*
France	2001–2004	-11.4 (-15.2, -7.4)*	2004–2017	-4.3 (-4.9, -3.7)*					-5.7 (-6.5, -4.9)*
Hungary	2000–2018	-4.8 (-5.6, -3.9)*							-4.8 (-5.6, -3.9)*
Ireland	2000–2008	-4.1 (-7.1, -1.0)*	2008–2018	-10.7 (-13.7, -7.5)*					-7.8 (-9.8, -5.7)*
Italy	2000–2008	-3.2 (-4.9, -1.6)*	2008–2012	-12.0 (-19.5, -3.7)*	2012–2018	-1.8 (-5.2, 1.8)			-4.8 (-6.8, -2.7)*
Netherlands	2000–2011	-5.9 (-7.3, -4.4)*	2011–2018	-0.3 (-3.9, 3.4)					-3.7 (-5.2, -2.2)*
Poland	2000–2018	-3.5 (-4.1, -2.8)*							-3.5 (-4.1, -2.8)*
Portugal	2000–2002	2.9 (-14.7, 24.2)	2002–2012	-11.6 (-13.8, -9.4)*	2012–2018	-0.7 (-7.2, 6.2)			-6.6 (-9.3, -3.7)*
Romania	2000–2003	-7.4 (-11.5, -3.0)*	2003–2008	-1.2 (-4.4, 2.1)	2008–2011	-9.1 (-19.5, 2.5)	2011–2018	-4.2 (-6.1, -2.3)*	-4.8 (-6.7, -2.8)*
Sweden	2000–2018	-3.6 (-5.6, -1.6)*							-3.6 (-5.6, -1.6)*
Slovakia	2000–2018	-3.6 (-4.4, -2.7)*							-3.6 (-4.4, -2.7)*
UK	2000–2008	-2.7 (-3.7, -1.7)*	2008–2012	-14.3 (-19.1, -9.2)*	2012–2018	1.9 (-0.3, 4.2)			-3.9 (-5.2, -2.6)*
**EU28**	**2001–2008**	-4.9 (-5.5, -4.2)*	**2008–2012**	-8.0 (-10.9, -5.1)*	**2012–2017**	-3.0 (-4.5, -1.4)*			-5.1 (-5.9, -4.3)*
**Accidents**									
Austria	2000–2018	-6.7 (-7.5, -5.9)*							-6.7 (-7.5, -5.9)*
Belgium	2003–2018	-6.5 (-7.8, -5.1)*							-6.5 (-7.8, -5.1)*
Bulgaria	2000–2008	0.4 (-2.2, 3.0)	2008–2011	-14.8 (-35.3, 12.4)	2011–2018	-0.6 (-5.0, 4.0)			-2.7 (-7.0, 1.8)
Switzerland	2000–2018	-5.0 (-6.1, -3.9)*							-5.0 (-6.1, -3.9)*
Czechia	2000–2018	-5.6 (-6.2, -4.9)*							-5.6 (-6.2, -4.9)*
Germany	2000–2018	-6.6 (-7.1, -6.2)*							-6.6 (-7.1, -6.2)*
Denmark	2000–2008	-4.3 (-6.7, -1.8)*	2008–2016	-13.0 (-17.0, -8.7)*	2016–2018	26.8 (-12.0, 82.8)			-5.3 (-9.2, -1.3)*
Greece	2000–2018	-4.4 (-5.9, -2.9)*							-4.4 (-5.9, -2.9)*
Spain	2000–2008	-6.3 (-8.2, -4.3)*	2008–2011	-22.6 (-40.5, 0.7)	2011–2018	-3.4 (-7.7, 1.1)			-8.1 (-12.0, -4.1)*
Finland	2000–2004	5.4 (-2.9, 14.5)	2004–2014	-9.3 (-11.8, -6.7)*	2014–2018	10.5 (-0.8, 23.2)			-2.0 (-4.9, 1.0)
France	2001–2004	-12.6 (-17.8, -7.1)*	2004–2017	-4.9 (-5.7, -4.1)*					-6.4 (-7.5, -5.3)*
Hungary	2000–2007	-2.2 (-4.3, 0.0)	2007–2010	-13.7 (-29.9, 6.2)	2010–2018	-4.6 (-7.3, -1.8)*			-5.3 (-8.4, -2.0)*
Ireland	2000–2018	-9.1 (-10.7, -7.3)*							-9.1 (-10.7, -7.3)*
Italy	2000–2007	-2.7 (-5.1, -0.2)*	2007–2014	-10.6 (-13.6, -7.5)*	2014–2018	0.6 (-7.1, 8.9)			-5.2 (-7.1, -3.2)*
Netherlands	2000–2012	-7.8 (-9.1, -6.5)*	2012–2018	-0.1 (-5.2, 5.4)					-5.3 (-7.0, -3.5)*
Poland	2000–2018	-4.1 (-4.8, -3.5)*							-4.1 (-4.8, -3.5)*
Portugal	2000–2002	22.1 (-5.9, 58.3)	2002–2012	-14.1 (-16.8, -11.4)*	2012–2018	1.7 (-7.1, 11.2)			-5.5 (-9.2, -1.6)*
Romania	2000–2003	-8.0 (-12.2, -3.5)*	2003–2008	-1.4 (-4.7, 2.0)	2008–2011	-10.3 (-20.9, 1.7)	2011–2018	-4.2 (-6.1, -2.2)*	-5.1 (-7.2, -3.0)*
Sweden	2000–2018	-6.6 (-9.5, -3.6)*							-6.6 (-9.5, -3.6)*
Slovakia	2000–2018	-4.1 (-5.1, -3.2)*							-4.1 (-5.1, -3.2)*
UK	2000–2007	-1.7 (-2.9, -0.4)*	2007–2012	-12.2 (-15.3, -9.0)*	2012–2018	-1.3 (-3.6, 1.1)			-4.6 (-5.8, -3.4)*
**EU28**	**2001–2008**	-5.4 (-6.2, -4.7)*	**2008–2013**	-9.1 (-11.3, -6.9)*	**2013–2017**	**-2.5 (-5.3, 0.4)**			-5.9 (-6.8, -5.0)*
**Transport accidents**									
Austria	2000–2018	-7.1 (-8.0, -6.1)*							-7.1 (-8.0, -6.1)*
Belgium	2003–2018	-7.9 (-9.0, -6.7)*							-7.9 (-9.0, -6.7)*
Bulgaria	2000–2008	4.3 (0.4, 8.3)*	2008–2012	-16.5 (-32.6, 3.5)	2012–2018	4.6 (-4.1, 14.1)			-0.6 (-5.6, 4.7)
Switzerland	2000–2018	-6.2 (-7.9, -4.5)*							-6.2 (-7.9, -4.5)*
Czechia	2000–2018	-5.7 (-6.5, -4.8)*							-5.7 (-6.5, -4.8)*
Germany	2000–2018	-7.8 (-8.2, -7.3)*							-7.8 (-8.2, -7.3)*
Denmark	2000–2009	-5.4 (-7.9, -2.9)*	2009–2015	-16.9 (-29.0, -2.9)*	2015–2018	8.1 (-16.8, 40.5)			-7.4 (-12.9, -1.6)*
Greece	2000–2012	-5.9 (-7.2, -4.6)*	2012–2015	18.4 (-15.4, 65.7)	2015–2018	-26.2 (-37.3, -13.0)*			-6.1 (-11.2, -0.7)*
Spain	2000–2007	-6.9 (-9.6, -4.2)*	2007–2014	-19.6 (-24.4, -14.4)*	2014–2018	8.5 (-7.7, 27.5)			-9.0 (-12.6, -5.3)*
Finland	2000–2007	-1.0 (-4.8, 3.1)	2007–2013	-10.8 (-17.8, -3.2)*	2013–2018	3.9 (-5.4, 14.2)			-3.0 (-6.5, 0.5)
France	2001–2004	-14.1 (-20.1, -7.7)*	2004–2017	-6.9 (-7.9, -5.9)*					-8.3 (-9.6, -6.9)*
Hungary	2000–2007	0.7 (-2.3, 3.9)	2007–2010	-19.0 (-39.6, 8.6)	2010–2018	-5.6 (-9.6, -1.5)*			-5.6 (-10.1, -1.0)*
Ireland	2000–2003	-17.6 (-29.9, -3.2)*	2003–2007	1.3 (-17.9, 25.1)	2007–2018	-15.1 (-18.9, -11.1)*			-12.1 (-16.7, -7.3)*
Italy	2000–2007	-2.9 (-4.4, -1.3)*	2007–2013	-12.8 (-15.4, -10.2)*	2013–2018	-2.9 (-6.4, 0.9)			-6.3 (-7.6, -5.0)*
Netherlands	2000–2018	-7.5 (-8.7, -6.2)*							-7.5 (-8.7, -6.2)*
Poland	2000–2018	-3.5 (-4.3, -2.7)*							-3.5 (-4.3, -2.7)*
Portugal	2000–2002	18.0 (-15.6, 65.1)	2002–2018	-12.1 (-13.9, -10.2)*					-9.1 (-12.5, -5.6)*
Romania	2000–2003	-9.6 (-16.1, -2.6)*	2003–2008	6.2 (1.1, 11.6)*	2008–2011	-12.7 (-26.9, 4.2)	2011–2018	-1.1 (-3.8, 1.6)	-2.7 (-5.6, 0.4)
Sweden	2000–2018	-7.5 (-8.9, -6.0)*							-7.5 (-8.9, -6.0)*
Slovakia	2000–2018	-5.1 (-6.4, -3.7)*							-5.1 (-6.4, -3.7)*
UK	2000–2008	-1.6 (-2.6, -0.7)*	2008–2011	-27.4 (-35.4, -18.4)*	2011–2018	-7.8 (-10.0, -5.6)*			-8.8 (-10.6, -7.0)*
**EU28**	**2001–2008**	-5.6 (-6.6, -4.6)*	**2008–2013**	-11.1 (-14.1, -8.1)*	**2013–2017**	**-3.1 (-7.1, 1.0)**			-6.8 (-8.0, -5.5)*
**Intentional self-harm**									
Austria	2000–2008	-7.3 (-10.6, -4.0)*	2008–2018	-0.6 (-3.6, 2.6)					-3.6 (-5.7, -1.5)*
Belgium	2003–2018	-2.4 (-4.0, -0.8)*							-2.4 (-4.0, -0.8)*
Bulgaria	2000–2018	-2.9 (-5.0, -0.7)*							-2.9 (-5.0, -0.7)*
Switzerland	2000–2018	-1.1 (-2.7, 0.5)							-1.1 (-2.7, 0.5)
Czechia	2000–2018	1.1 (-0.3, 2.5)							1.1 (-0.3, 2.5)
Germany	2000–2006	-5.9 (-9.0, -2.7)*	2006–2018	0.5 (-0.8, 1.9)					-1.7 (-2.9, -0.4)*
Denmark	2000–2006	-8.8 (-16.1, -0.8)*	2006–2018	2.1 (-1.5, 5.8)					-1.7 (-4.8, 1.6)
Greece	2000–2002	-26.6 (-40.8, -8.9)*	2002–2018	2.2 (0.7, 3.6)*					-1.5 (-3.4, 0.4)
Spain	2000–2010	-4.6 (-7.6, -1.5)*	2010–2018	7.0 (2.3, 12.0)*					0.4 (-2.0, 2.9)
Finland	2000–2018	-1.5 (-3.0, -0.1)*							-1.5 (-3.0, -0.1)*
France	2001–2007	-4.1 (-6.3, -1.8)*	2007–2010	6.5 (-7.6, 22.8)	2010–2017	-6.9 (-8.8, -4.9)*			-3.4 (-5.9, -0.9)*
Hungary	2000–2018	-2.4 (-3.8, -1.1)*							-2.4 (-3.8, -1.1)*
Ireland	2000–2018	-3.0 (-5.0, -0.9)*							-3.0 (-5.0, -0.9)*
Italy	2000–2018	-0.0 (-1.1, 1.1)							-0.0 (-1.1, 1.1)
Netherlands	2000–2018	2.1 (0.6, 3.6)*							2.1 (0.6, 3.6)*
Poland	2000–2014	0.0 (-1.2, 1.2)	2014–2018	-10.8 (-20.4, -0.1)*					-2.5 (-4.9, -0.1)*
Portugal	2000–2017	-0.6 (-2.7, 1.5)							-0.6 (-2.7, 1.5)
Romania	2000–2018	-0.6 (-1.9, 0.7)							-0.6 (-1.9, 0.7)
Sweden	2000–2018	0.8 (-0.7, 2.2)							0.8 (-0.7, 2.2)
Slovakia	2000–2018	-0.5 (-2.2, 1.1)							-0.5 (-2.2, 1.1)
UK	2000–2003	-11.9 (-16.0, -7.5)*	2003–2014	0.6 (-0.3, 1.4)	2014–2018	14.4 (11.2, 17.6)*			1.2 (0.2, 2.3)*
**EU28**	**2001–2017**	-1.4 (-1.8, -1.0)*							-1.4 (-1.8, -1.0)*
**Poisoning**									
Austria	2000–2001	-16.8							-16.8
Belgium	2004–2009	-2.3 (-15.0, 12.2)							-2.3 (-15.0, 12.2)
Bulgaria	2000–2005	-14.7 (-24.0, -4.1)*							-14.7 (-24.0, -4.1)*
Czechia	2000–2014	1.5 (-0.9, 3.9)							1.5 (-0.9, 3.9)
Germany	2000–2014	-5.9 (-8.7, -3.0)*	2014–2018	19.3 (-3.0, 46.9)					-0.8 (-5.3, 4.0)
Greece	2000–2010	-11.4 (-19.6, -2.4)*							-11.4 (-19.6, -2.4)*
Spain	2000–2016	-7.2 (-9.6, -4.8)*							-7.2 (-9.6, -4.8)*
Finland	2000–2012	-0.5 (-4.1, 3.2)	2012–2018	11.9 (-21.4, 59.4)					3.5 (-6.4, 14.3)
France	2001–2003	-14.4 (-58.4, 75.8)	2003–2006	37.9 (-25.1, 153.8)	2006–2009	-27.6 (-59.9, 30.8)	2009–2017	-1.0 (-9.3, 8.0)	-2.5 (-16.1, 13.4)
Hungary	2003–2013	-2.4 (-14.8, 11.8)							-2.4 (-14.8, 11.8)
Ireland	2002–2007	6.7 (-68.5, 261.6)							6.7 (-68.5, 261.6)
Italy	2001–2015	0.8 (-2.6, 4.3)							0.8 (-2.6, 4.3)
Poland	2000–2016	-5.1 (-7.5, -2.6)*	2016–2018	46.1 (-28.2, 197.5)					-0.4 (-7.6, 7.3)
Romania	2000–2018	-9.2 (-10.5, -8.0)*							-9.2 (-10.5, -8.0)*
Sweden	2001–2018	1.3 (-1.3, 3.8)							1.3 (-1.3, 3.8)
UK	2000–2012	-2.0 (-4.2, 0.3)	2012–2018	8.7 (2.2, 15.6)*					1.4 (-0.9, 3.8)
**EU28**	**2001–2012**	-7.3 (-8.5, -6.2)*	**2012–2017**	**-0.0 (-5.1, 5.4)**					-5.1 (-6.7, -3.5)*
**Assault**									
Austria	2000–2006	-1.7 (-9.2, 6.5)							-1.7 (-9.2, 6.5)
Belgium	2003–2018	-5.5 (-8.0, -3.0)*							-5.5 (-8.0, -3.0)*
Bulgaria	2000–2009	-5.4 (-10.0, -0.6)*							-5.4 (-10.0, -0.6)*
Switzerland	2000–2005	12.6 (11.6, 13.6)*							12.6 (11.6, 13.6)*
Czechia	2000–2013	-1.8 (-7.2, 4.0)							-1.8 (-7.2, 4.0)
Germany	2000–2018	-2.3 (-3.7, -0.9)*							-2.3 (-3.7, -0.9)*
Denmark	2000–2017	-0.1 (-4.7, 4.7)							-0.1 (-4.7, 4.7)
Spain	2000–2018	-1.1 (-3.7, 1.6)							-1.1 (-3.7, 1.6)
Finland	2000–2012	-1.4 (-3.4, 0.6)							-1.4 (-3.4, 0.6)
France	2001–2017	-4.1 (-5.7, -2.5)*							-4.1 (-5.7, -2.5)*
Hungary	2000–2018	-2.3 (-5.3, 0.7)							-2.3 (-5.3, 0.7)
Italy	2000–2018	-3.0 (-4.9, -1.1)*							-3.0 (-4.9, -1.1)*
Netherlands	2000–2018	-2.9 (-4.7, -1.1)*							-2.9 (-4.7, -1.1)*
Poland	2000–2018	-6.0 (-7.2, -4.8)*							-6.0 (-7.2, -4.8)*
Portugal	2001–2006	14.7 (7.1, 22.9)*							14.7 (7.1, 22.9)*
Romania	2000–2018	-6.0 (-7.5, -4.5)*							-6.0 (-7.5, -4.5)*
Sweden	2000–2017	0.3 (-1.5, 2.1)							0.3 (-1.5, 2.1)
Slovakia	2000–2004	7.1 (-25.7, 54.2)							7.1 (-25.7, 54.2)
UK	2000–2002	-31.5 (-50.9, -4.6)*	2002–2014	-6.8 (-9.9, -3.6)*					-10.8 (-15.0, -6.4)*
**EU28**	**2001–2017**	-4.9 (-5.4, -4.4)*							-4.9 (-5.4, -4.4)*

*APC* annual percent change, *AAPC* average annual percent change

^*^significantly different from 0 (*p* < 0.05)

The overall trend in mortality from non-natural causes decreased from 2000 to 2018 for all countries except Bulgaria, where the decreasing trend was not statistically significant. Rates for the EU28 declined by 5.1% per year over the entire period. The largest declines were in Ireland (AAPC -7.8), Spain (AAPC -6.7) and Portugal (AAPC -6.6). Smaller declines were observed in Eastern European countries such as Bulgaria (-2.7), the Czech Republic (-3.4), Poland (-3.5), Slovakia (-3.6) and Finland (-3.6). In the EU28, the decline in mortality was more pronounced between 2008 and 2012 (APC -8.0). A similar trend was observed for many European countries, with France and Germany showing greater decline in the first period (2001–2004 and 2000–2007, respectively). Some other countries also showed a log-linear trend over the entire period (Austria, Belgium, Greece, Switzerland, Sweden, Finland, Slovakia, and Poland).

The mortality rates for accidental events decreased by 5.9% per year in the EU as a whole. The largest decreases since 2000 were in Ireland (AAPC -9.1%) and Austria (AAPC -6.7%). Finland and Bulgaria also experienced declines but the AAPCs estimates do not reach statistical significance. The strongest downward significant trend was observed in Denmark between 2008 and 2016 (-13.0%).

Throughout the investigated period, traffic fatalities rates decreased by 6.8% per year. The largest decrease since 2000 was observed in Ireland (-12.1%), Portugal and Spain (-9.0%), and the United Kingdom (-8.8%). In Bulgaria (-0.6%), Romania (-2.7) and Finland (-3.0), the decline was limited and not statistically significant. While Finland showed greater decrease (-10.8%) between 2007 and 2013 and stability in other periods, Bulgaria and Romania showed increasing rates in 2000–2008 (Bulgaria, 4.3%) and in 2003–2008 (Romania, 6.2%). The largest significant decreases were observed in the UK (27.4%) in 2008–2011, Greece (-26.2%) in 2015–2018, Spain (-19.6) in 2007–2014 and Denmark (-16.9) in 2009–2015.

Throughout the assessed period, rates of intentional self-harm declined slightly, with an AAPC of -1.4 in the EU. However, rates increased throughout the same period in some countries, such as the Netherlands (2.1%) and the United Kingdom (1.2%), or remained stable (i.e. the Czech Republic, Spain, Sweden, Romania, Romania, Portugal, Italy, Greece, Denmark, Switzerland). While the same upward trend was observed in the Netherlands throughout the same period, a downward trend was first observed in the United Kingdom (2000–2003), followed by a long period of stability (2003–2014) and a strong upward trend in recent years (2014–2018).

Data on poisonings and assaults were limited and not available for all countries, and only countries with at least six years of observation between 2000 and 2018 were included. In the EU, a decreasing trend in poisoning rates was observed between 2001 and 2012 (-7.3%), while rates remained stable in subsequent years. In particular, the largest significant decrease since 2000 was observed in Bulgaria (-14.7%), Greece (-11.4%), Romania (-9.2%) and Spain (-7.2%).

Throughout the period, the EU assault mortality rate declined by 4.9% per year. The largest decrease per year since 2000 was observed in the United Kingdom (-10.8%), Romania, and Poland (-6.0), Bulgaria (-5.4%) and Belgium (since 2003, -5.50).

Supplementary Table 1 provides the AAPCs and trends for each cause by sex. Throughout the period, mortality rates from external causes declined similarly for boys and girls ( -5.2% versus -4.9%). However, in some countries (Bulgaria, Denmark and Finland), the decline was greater for girls than for boys. Similar trends were observed for mortality from accidental events and transport accidents. The mortality rate for intentional self-inflicted injuries decreased for boys (-2.1), whereas a slight increase was observed for girls (0.6). Large trend differences were observed between boys and girls, and even when a decrease was observed in both sexes, the trend was more pronounced in boys than in girls.

## Discussion

As far as we are aware, few articles have been published in recent years that systematically examine trends in non-natural causes of mortality in children and adolescents in Europe [32, 33]. Other studies evaluate specific causes of death at the national level and refer to more distant years [32, 34–37]. This study, therefore, aims to fill at least part of this gap by presenting a recent (in the last two decades) and cross-national descriptive analysis of this phenomenon in several European countries.

It has been noted that in Europe, mortality among children and adolescents due to non-natural causes has decreased significantly over the last two decades. However, geographic disparities continue to be observed. As with other causes of death in young people (e.g. cancer, infectious diseases), geographic disparities are multifactorial and include health system performance; nevertheless, cultural and socioeconomic characteristics may play a more important role in non-natural causes of death [38]. Specifically, such cultural and socioeconomic characteristics, including stigma and difficulty in recognizing violent intent, may be highly relevant in coding mortality related to external [39, 40] causes. Throughout the period, Spain, Ireland and Portugal experienced the largest declines in mortality rates due to non-natural causes. Poland, the Czech Republic, Slovakia and Romania recorded sharp declines in mortality rates due to non-natural causes, but are still above the European average. Conversely, Bulgaria had the highest mortality rates due to non-natural causes in 2003–2007 and recorded a decline in AAPC that was below the EU average and not in line with other European countries. In Western countries, mortality from non-natural causes was already declining before 2000 [32], and in some of these countries (Austria, Belgium, Finland, France, Germany, Italy, the Netherlands, Switzerland) rates have continue to decline, whereas in others (Spain, Portugal, the United Kingdom, Denmark) they have remained stable since 2010. This suggests that attention to injury prevention and safety regulations continues to be an effective measure to prevent injury-related deaths [41, 42].

The greatest decrease in mortality was recorded in accidental events and transport accidents. These trends were also observed in other studies in Northern Europe [34] and other European countries [32, 36, 37]. Although the United Kingdom and Sweden had lower rates from 2003 to 2007, there was a continuous decline in mortality rates. This long-lasting decrease can be attributed to concrete efforts to reduce transport accidents [43, 44], through the implementation of policies and legislation that have increased the use of child safety belts, developed safer road designs, and improved vehicle safety [41, 42, 45].

As reported in another study [46], mortality rates for intentional self-inflicted injuries were higher in Northern countries (i.e. Finland, Ireland, Poland) in 2003–2007, and rates were higher in males [46]. Since 2001, rates have declined across the EU28, particularly among boys; however, rates remain higher in Finland and Poland than in other countries. Of note, rates have increased in the Netherlands and the Unite Kingdom, particularly among girls. Mortality from Intentional self-harm can be prevented by implementing prevention strategies, such as limiting access to commonly used lethal means (including medications) and promoting access to mental health and other services [47]. However, most countries, do not have such measures [48]. Self-poisoning with medications is a common method of suicide in many European countries, and prohibiting access to medications commonly used in suicides has been shown to be effective in preventing suicides [47]. In Italy, for example, the use of opioid analgesics remain much lower than in Northern Europe [49]. In addition numerous efforts have been made to ensure that physician and pharmacists exercise greater vigilance in prescribing or dispensing opioids to high-risk patients [50].

Poisoning rates in the EU28 have declined throughout the period. While rates were stable in most countries, a rapid decline was observed in Bulgaria, Romania, Greece, and Spain. Despite the decrease observed throughout the study period, poisoning rates in Romania remained high compared to the other countries. It should be recalled that accidental poisonings, especially among young children, could be prevented by improved prevention measures [48, 51] (i.e. proper packaging of medicines and household products, education on the importance of keeping such products safely out of the reach of children).

Aggression rates have also declined in the EU28 over this period. In Belgium, Bulgaria and Romania, rates have declined significantly. In Switzerland and Portugal, rates appear to be increasing, but there are few observations at any one time to draw conclusions.

The differences found between countries may have several causes, such as social, cultural, and health reasons [22, 38]. Above all, the type of health care system plays a crucial role, as does the number of autopsies performed. This last aspect is perhaps the one that deserves more attention and consideration. Autopsy is, in fact, the instrument that allows the most truthful death certifications possible [52]. Although it is impossible to determine the number of forensic autopsies requested in each country, the general impression is that in Europe interest in violent and suspicious deaths is decreasing. This has been reported in several articles, involving the Netherlands, Norway, Denmark, Germany, Austria, and Italy as well [22, 53–55]. However, it should never be underestimated that through forensic autopsies it is possible to control death and disease due to non-natural causes, hence protecting public health in its broadest sense. They allow monitoring of the epidemiology of lethal violence in the population and are the last bastion to protect the rights of the deceased and their families when clinical and preventive efforts have failed [22]. Since violence can be lethal [56]and has serious consequences for the physical and mental health of survivors, it would be appropriate to start considering it like any other disease and face it as such [57]. In addition, large-scale routine autopsy studies, e.g., in toxicology and microbiology, can help identify emerging social trends in deaths and diseases related to drug abuse [58], poisoning [59], infection [60], and even environmental pollution [61], providing important information for the development of prevention strategies. Finally, autopsy also allows the correct classification of suicide cases, whose trend, unlike other non-natural deaths, is the only one in the population that is not significantly decreasing, but actually increasing in some countries [62]. All this is true for the whole population and even more so for one of the main risk groups, namely children and adolescents [63].

### Strengths and limitations

Several factors must be considered when interpreting these results. A strength of this study is that is based on official national sources, and we have limited the analysis to countries with satisfactory coverage of death certificates and a meaningful number of cases; nevertheless, some degree of misclassification is possible, as is random variation due to small numbers of deaths. In addition, incidence may be affected by changes in registration options and criteria between countries and calendar period. Finally, the analysis is limited to mortality, so data on nonfatal injuries were not included.

Further studies based on specific country variables are needed to fully understand the observed trend, such as traffic and vehicle safety measures [46], child abuse and poisoning prevention measures, socioeconomic factors, and education levels in each country. However, the descriptive analysis presented in this study can serve as a starting point for future, more focused assessments by working groups in individual countries to identify appropriate prevention measures. In the medium term, another descriptive study will be essential to assess whether the trend of non-natural deaths in children and adolescents has changed as a result of the recent Covid-19 pandemic [47–49], which is known to have had a tremendous impact on the mental and physical health of billions of people. Preliminary cross-national studies do not appear to show an increase in suicides in this context [64], but this finding needs further investigation.

## Conclusion

This study found that mortality among children and adolescents from non-natural causes decreased significantly in Europe over an 18-year period, although geographic differences between countries remain. Furthermore, despite this decrease, there are still European countries where the number of non-natural deaths is higher than the European average. These findings have only begun to shed light on the hidden world of non-natural deaths among children and adolescents, but they clearly show that their further reduction is still possible in many countries, and should encourage the improvement of measures to prevent child mortality as much as possible.

### Supplementary Information

**Additional file 1: Table S1.** Joinpoint results by external causes 2000-2019 by gender.

## Data Availability

The data that support the findings of this study are openly available in https://ec.europa.eu/eurostat/data/database.

## References

[1] UNICEF. Child Mortality, Report 2018. Available from: https://data.unicef.org/.

[2] Our World in Data. Our World Data. Cited 2022 Feb 23. Available from: https://ourworldindata.org.

[3] Kyu HH, Stein CE, Boschi Pinto C (2018). Causes of death among children aged 5–14 years in the WHO European Region: a systematic analysis for the Global Burden of Disease Study 2016. Lancet Child Adolesc Health.

[4] Harris A (2017). ‘Natural’ and ‘Unnatural’ medical deaths and coronial law: A UK and international review of the medical literature on natural and unnatural death and how it applies to medical death certification and reporting deaths to coroners: natural/unnatural death: a scientific review. Med Sci Law.

[5] Transforming our World: The 2030 Agenda for Sustainable Development | Department of Economic and Social Affairs. Cited 2022 Feb 23. Available from: https://sdgs.un.org/publications/transforming-our-world-2030-agenda-sustainable-development-17981.

[6] Wu Y, Deng Y, Wei B (2022). Global, regional, and national childhood cancer burden, 1990–2019: an analysis based on the Global Burden of Disease Study 2019. J Adv Res.

[7] Bertuccio P, Alicandro G, Malvezzi M (2020). Childhood cancer mortality trends in Europe, 1990–2017, with focus on geographic differences. Cancer Epidemiol.

[8] Cunningham RM, Walton MA, Carter PM (2018). The major causes of death in children and adolescents in the United States. N Engl J Med.

[9] Escobar MA, Pflugeisen BM, Duralde Y (2016). Development of a systematic protocol to identify victims of non-accidental trauma. Pediatr Surg Int.

[10] Martrille L, Cattaneo C, Dorandeu A, Baccino E (2006). A multicentre and prospective study of suspected cases of child physical abuse. Int J Legal Med.

[11] Palusci VJ, Wirtz SJ, Covington TM (2010). Using capture-recapture methods to better ascertain the incidence of fatal child maltreatment. Child Abuse Negl.

[12] Crume TL, DiGuiseppi C, Byers T, Sirotnak AP, Garrett CJ (2002). Underascertainment of child maltreatment fatalities by death certificates, 1990–1998. Pediatrics.

[13] Sauvageau A, Racette S (2008). Child and adolescent victims in forensic autopsy: a 5-year retrospective study. J Forensic Sci.

[14] Fadel SA, Amouzou A (2022). Child and adolescent deaths: a call for strengthening mortality surveillance systems. Lancet Glob Health.

[15] Bundock EA, Corey TS, The National Association of Medical Examiners’ Panel on Sudden Unexpected Death in Pediatrics (2019). Unexplained pediatric deaths: investigation, certification, and family needs.

[16] Otieno P, Akelo V, Khagayi S (2023). Acceptability of minimally invasive autopsy by community members and healthcare workers in Siaya and Kisumu counties, western Kenya, 2017–2018. PLoS Glob Public Health.

[17] Barbara G, Collini F, Buggio L, et al. An Italian single-centre retrospective analysis of 1106 consecutive cases of child and adolescent abuse: key elements of effective practices. Minerva Pediatr. 2021;4(3):325–31. 10.23736/S2724-5276.21.06459-4 34184469

[18] Reynders A, Scheerder G, Van Audenhove C (2011). The reliability of suicide rates: an analysis of railway suicides from two sources in fifteen European countries. J Affect Disord.

[19] Taggi F, Marturano P, Macchia T (2009). The urgency of establishing a rapid monitoring system for mortality due to traffic accidents (as well as for all mortality due to violence and accidents). Ann Ig Med Prev E Comunita.

[20] Fracasso T (2021). Selected abstracts from the 25th congress of the international academy of legal medicine. Int J Legal Med.

[21] Stefano Tambuzzi, Graziano Crudele, Lidia Maggioni, Cristina Cattaneo. Autopsies on minors: a taboo? The experience of Milan (2000-2020). Int J Legal Med. 2021:11–119. Cited 2023 Oct 4. Available from: 10.1007/s00414-021-02613-z. 10.1007/s00414-023-03114-x PMC10861724 37934209

[22] Cattaneo C, Tambuzzi S, Maggioni L, Zoja R (2022). Has violent death lost the interest of epidemiology?. Int J Epidemiol.

[23] Home - Eurostat. Cited 2022 Feb 23. Available from: https://ec.europa.eu/eurostat/en/.

[24] ICD - ICD-10 - International Classification of Diseases, Tenth Revision. 2021. Cited 2022 Feb 23. Available from: https://www.cdc.gov/nchs/icd/icd10.htm.

[25] Doll R, Smith PG. Comparison between registries: age-standardized rates. Cancer Incidence in Five Continents. IARC Scientific Publications; Lyon: International Agency for Research on Cancer; 1992. p. 865–870. 1284608

[26] Joinpoint Regression Program. Cited 2022 Mar 17. Available from: https://surveillance.cancer.gov/joinpoint/.

[27] Kim HJ, Fay MP, Feuer EJ, Midthune DN (2000). Permutation tests for joinpoint regression with applications to cancer rates. Stat Med.

[28] Lerman PM (1980). Fitting segmented regression models by grid search. J R Stat Soc Ser C Appl Stat.

[29] Clegg LX, Hankey BF, Tiwari R, Feuer EJ, Edwards BK (2009). Estimating average annual per cent change in trend analysis. Stat Med.

[30] R Core Team. R: A Language and Environment for Statistical Computing. Vienna: R Foundation for Statistical Computing; 2022.

[31] Xu J, Lin Y, Yang M, Zhang L (2020). Statistics and pitfalls of trend analysis in cancer research: a review focused on statistical packages. J Cancer.

[32] Armour-Marshall J, Wolfe I, Richardson E, Karanikolos M, McKee M (2012). Childhood deaths from injuries: trends and inequalities in Europe. Eur J Public Health.

[33] Sethi D, Aldridge E, Rakovac I, Makhija A (2017). Worsening inequalities in child injury deaths in the WHO European Region. Int J Environ Res Public Health.

[34] Otterman G, Lahne K, Arkema EV, Lucas S, Janson S, Hellström-Westas L (2019). Childhood death rates declined in Sweden from 2000 to 2014 but deaths from external causes were not always investigated. Acta Paediatr Oslo Nor 1992.

[35] Bäckström D, Steinvall I, Sjöberg F (2017). Change in child mortality patterns after injuries in Sweden: a nationwide 14-year study. Eur J Trauma Emerg Surg Off Publ Eur Trauma Soc.

[36] Grajda A, Kułaga Z, Gurzkowska B, Góźdź M, Wojtyło M, Litwin M (2017). Trends in external causes of child and adolescent mortality in Poland, 1999–2012. Int J Public Health.

[37] Gijzen S, Boere-Boonekamp MM, L’Hoir MP, Need A (2014). Child mortality in the Netherlands in the past decades: an overview of external causes and the role of public health policy. J Public Health Policy.

[38] Hardelid P, Davey J, Dattani N, Gilbert R, Working Group of the Research and Policy Directorate of the Royal College of Paediatrics and Child Health (2013). Child deaths due to injury in the four UK countries: a time trends study from 1980 to 2010. PloS One..

[39] McCarroll JE, Fisher JE, Cozza SJ, Whalen RJ (2021). Child maltreatment fatality review: purposes, processes, outcomes, and challenges. Trauma Violence Abuse.

[40] Lu TH, Walker S, Anderson RN, McKenzie K, Bjorkenstam C, Hou WH (2007). Proportion of injury deaths with unspecified external cause codes: a comparison of Australia, Sweden, Taiwan and the US. Inj Prev J Int Soc Child Adolesc Inj Prev.

[41] James SL, Castle CD, Dingels ZV (2020). Global injury morbidity and mortality from 1990 to 2017: results from the Global Burden of Disease Study 2017. Inj Prev.

[42] Preventing injuries and violence: an overview. World Health Organization; 2022. Available from: https://iris.who.int/bitstream/handle/10665/361331/9789240047136-eng.pdf?sequence=1.

[43] Reducing unintentional injuries on the roads among children and young people. :26.

[44] Steinbach R, Cairns J, Grundy C, Edwards P (2013). Cost benefit analysis of 20 mph zones in London. Inj Prev J Int Soc Child Adolesc Inj Prev.

[45] Children in road traffic. DaCoTA. Available from: https://road-safety.transport.ec.europa.eu/system/files/2021-07/01-child_traffic_safety_en.pdf.

[46] Junuzovic M, Lind KMT, Jakobsson U (2022). Child suicides in Sweden, 2000–2018. Eur J Pediatr.

[47] Preventing suicide: A global imperative. Cited 2023 Jan 9. Available from: https://www.who.int/publications-detail-redirect/9789241564779.

[48] Nistor N, Frasinariu OE, Rugină A, Ciomaga IM, Jităreanu C, Ştreangă V (2018). Epidemiological study on accidental poisonings in children from northeast romania. Medicine (Baltimore).

[49] Bosetti C, Santucci C, Radrezza S, Erthal J, Berterame S, Corli O (2019). Trends in the consumption of opioids for the treatment of severe pain in Europe, 1990–2016. Eur J Pain Lond Engl.

[50] Gentile G, Tambuzzi S, Calati R, Zoja R (2022). A descriptive cohort of suicidal cancer patients: analysis of the autopsy case series from 1993 to 2019 in Milan (Italy). Int J Environ Res Public Health.

[51] Schwebel DC, Evans WD, Hoeffler SE (2017). Unintentional child poisoning risk: A review of causal factors and prevention studies. Child Health Care..

[52] Rosendahl A, Mjörnheim B, Eriksson LC (2021). Autopsies and quality of cause of death diagnoses. SAGE Open Med.

[53] Blokker BM, Weustink AC, Hunink MGM, Oosterhuis JW (2017). Autopsy rates in the Netherlands: 35 years of decline. PLoS One.

[54] Gaensbacher S, Waldhoer T, Berzlanovich A (2012). The slow death of autopsies: a retrospective analysis of the autopsy prevalence rate in Austria from 1990 to 2009. Eur J Epidemiol.

[55] Frost J, Slørdal L, Vege Å, Nordrum IS (2012). Forensic autopsies in a naturalistic setting in Norway: autopsy rates and toxicological findings. Forensic Sci Int.

[56] Biehler-Gomez L, Maggioni L, Tambuzzi S, Kustermann A, Cattaneo C (2022). Twenty years of femicide in Milan: a retrospective medicolegal analysis. Sci Justice J Forensic Sci Soc.

[57] Cattaneo C, Tambuzzi S, De Vecchi S, Maggioni L, Costantino G. Consequences of the lack of clinical forensic medicine in emergency departments. Int J Legal Med. 2023. *In press*, Available from: 10.1007/s00414-023-02973-8. 10.1007/s00414-023-02973-8 PMC10772006 36806756

[58] Ciccarone D (2021). The rise of illicit fentanyls, stimulants and the fourth wave of the opioid overdose crisis. Curr Opin Psychiatry.

[59] Tambuzzi S, Vacchiano L, Gentile G, Boracchi M, Zoja R, Migliorini AS. A Forensic Case of Suicide Ingestion of Paraquat Herbicide: New Histological Insights and Revision of the Literature. Am J Forensic Med Pathol. 2023. *In press*. 10.1097/PAF.0000000000000878. 10.1097/PAF.0000000000000878 PMC11446524 37728953

[60] Salerno M, Sessa F, Piscopo A (2020). No autopsies on COVID-19 deaths: a missed opportunity and the lockdown of science. J Clin Med.

[61] da Motta Singer J, de André CD, de André PA (2023). Assessing socioeconomic bias of exposure to urban air pollution: an autopsy-based study in São Paulo Brazil. Lancet Reg Health Am..

[62] Suicide rising across the US. Centers for Disease Control and Prevention. Available from: https://www.cdc.gov/vitalsigns/pdf/vs-0618-suicide-H.pdf.

[63] Ruch DA, Sheftall AH, Schlagbaum P, Rausch J, Campo JV, Bridge JA (2019). Trends in suicide among youth aged 10 to 19 years in the United States, 1975 to 2016. JAMA Netw Open.

[64] Pirkis J, Gunnell D, Shin S (2022). Suicide numbers during the first 9–15 months of the COVID-19 pandemic compared with pre-existing trends: an interrupted time series analysis in 33 countries. EClinicalMedicine.

