# Supplementary material for: Trends in mortality from non-natural causes in children and adolescents (0–19 years) in Europe from 2000 to 2018
Source: BMC Public Health. 2023 Nov 10;23:2223. doi: 10.1186/s12889-023-17040-5 (PMC10638782; doi:10.1186/s12889-023-17040-5)
Supplement: Supplementary file 1 — Additional file 1: Table S1. Joinpoint results by external causes 2000-2019 by gender. [file 12889_2023_17040_MOESM1_ESM.docx]

Table 1. *Joinpoint* results by external causes 2000-2019 by gender

Supplementary Table 1: boys

| **Country** | **Trend 1** | **APC 1** | **Trend 2** | **APC 2** | **Trend 3** | **APC 3** | **Trend 4** | **APC 4** | **AAPC** |
| --- | --- | --- | --- | --- | --- | --- | --- | --- | --- |
| **External causes** | | | | | | | | | |
| Austria | 2000-2018 | -4.9 (-5.6 ,-4.1)* |  |  |  |  |  |  | -4.9 (-5.6 ,-4.1)* |
| Belgium | 2003-2018 | -4.9 (-5.9 ,-3.9)* |  |  |  |  |  |  | -4.9 (-5.9 ,-3.9)* |
| Bulgaria | 2000-2018 | -2.7 (-3.6 ,-1.8)* |  |  |  |  |  |  | -2.7 (-3.6 ,-1.8)* |
| Czechia | 2000-2018 | -4.1 (-4.8 ,-3.4)* |  |  |  |  |  |  | -4.1 (-4.8 ,-3.4)* |
| Denmark | 2000-2011 | -4.8 (-7.0 ,-2.6)* | 2011-2016 | -14.4 (-25.2 ,-2.0)* | 2016-2018 | 41.8 (-3.0 ,107.4) |  |  | -3.4 (-8.2 ,1.7) |
| Finland | 2000-2004 | 3.5 (-5.5 ,13.3) | 2004-2015 | -6.8 (-9.3 ,-4.3)* | 2015-2018 | 12.7 (-7.1 ,36.7) |  |  | -1.5 (-5.1 ,2.1) |
| France | 2001-2004 | -10.4 (-15.0 ,-5.6)* | 2004-2017 | -4.8 (-5.5 ,-4.1)* |  |  |  |  | -5.9 (-6.8 ,-4.9)* |
| Germany | 2000-2007 | -7.1 (-8.1 ,-6.1)* | 2007-2018 | -4.2 (-4.9 ,-3.5)* |  |  |  |  | -5.3 (-5.9 ,-4.8)* |
| Greece | 2000-2018 | -4.2 (-5.1 ,-3.3)* |  |  |  |  |  |  | -4.2 (-5.1 ,-3.3)* |
| Hungary | 2000-2007 | -1.2 (-4.4 ,2.0) | 2007-2010 | -14.3 (-35.8 ,14.3) | 2010-2018 | -1.6 (-5.4 ,2.3) |  |  | -3.7 (-8.1 ,0.9) |
| Ireland | 2000-2008 | -3.7 (-7.1 ,-0.2)* | 2008-2018 | -10.8 (-14.3 ,-7.3)* |  |  |  |  | -7.7 (-10.0 ,-5.5)* |
| Italy | 2000-2007 | -3.0 (-5.8 ,-0.1)* | 2007-2014 | -9.4 (-12.8 ,-5.8)* | 2014-2018 | 1.9 (-6.4 ,10.9) |  |  | -4.5 (-6.7 ,-2.3)* |
| Netherlands | 2000-2008 | -6.9 (-9.1 ,-4.7)* | 2008-2018 | -2.1 (-4.1 ,-0.1)* |  |  |  |  | -4.3 (-5.6 ,-2.9)* |
| Poland | 2000-2018 | -3.4 (-4.0 ,-2.8)* |  |  |  |  |  |  | -3.4 (-4.0 ,-2.8)* |
| Portugal | 2000-2018 | -8.7 (-10.0 ,-7.3)* |  |  |  |  |  |  | -8.7 (-10.0 ,-7.3)* |
| Romania | 2000-2018 | -4.3 (-4.9 ,-3.8)* |  |  |  |  |  |  | -4.3 (-4.9 ,-3.8)* |
| Slovakia | 2000-2018 | -3.6 (-4.5 ,-2.6)* |  |  |  |  |  |  | -3.6 (-4.5 ,-2.6)* |
| Spain | 2000-2005 | -3.6 (-7.3 ,0.3) | 2005-2014 | -12.3 (-14.8 ,-9.8)* | 2014-2018 | 4.2 (-6.0 ,15.5) |  |  | -6.5 (-8.9 ,-4.0)* |
| Sweden | 2000-2018 | -3.5 (-5.3 ,-1.8)* |  |  |  |  |  |  | -3.5 (-5.3 ,-1.8)* |
| Switzerland | 2000-2018 | -4.1 (-5.2 ,-2.9)* |  |  |  |  |  |  | -4.1 (-5.2 ,-2.9)* |
| UK | 2000-2008 | -2.9 (-4.0 ,-1.8)* | 2008-2012 | -13.9 (-19.4 ,-8.1)* | 2012-2018 | 1.1 (-1.4 ,3.7) |  |  | -4.2 (-5.7 ,-2.7)* |
| **EU** | **2001-2008** | **-4.8 (-5.6 ,-4.1)*** | **2008-2012** | **-8.1 (-11.5 ,-4.7)*** | **2012-2017** | **-3.2 (-5.1 ,-1.3)*** |  |  | **-5.2 (-6.1 ,-4.2)*** |
| **Accidents** | | | | | | | | | |
| Austria | 2000-2018 | -6.5 (-7.4 ,-5.7)* |  |  |  |  |  |  | -6.5 (-7.4 ,-5.7)* |
| Belgium | 2003-2018 | -6.8 (-8.0 ,-5.5)* |  |  |  |  |  |  | -6.8 (-8.0 ,-5.5)* |
| Bulgaria | 2000-2008 | 1.2 (-1.5 ,3.9) | 2008-2011 | -13.9 (-35.0 ,14.1) | 2011-2018 | 0.2 (-4.2 ,4.8) |  |  | -1.9 (-6.3 ,2.7) |
| Czechia | 2000-2018 | -5.3 (-6.1 ,-4.5)* |  |  |  |  |  |  | -5.3 (-6.1 ,-4.5)* |
| Denmark | 2000-2008 | -3.8 (-6.9 ,-0.7)* | 2008-2016 | -13.9 (-19.2 ,-8.2)* | 2016-2018 | 44.3 (-6.1 ,121.8) |  |  | -4.2 (-8.9 ,0.7) |
| Finland | 2000-2004 | 4.9 (-4.3 ,14.9) | 2004-2014 | -9.4 (-12.2 ,-6.5)* | 2014-2018 | 13.3 (0.4 ,27.9)* |  |  | -1.6 (-4.9 ,1.8) |
| France | 2001-2004 | -11.7 (-16.9 ,-6.1)* | 2004-2017 | -5.3 (-6.1 ,-4.5)* |  |  |  |  | -6.5 (-7.7 ,-5.4)* |
| Germany | 2000-2018 | -6.6 (-7.1 ,-6.1)* |  |  |  |  |  |  | -6.6 (-7.1 ,-6.1)* |
| Greece | 2000-2018 | -4.6 (-5.6 ,-3.6)* |  |  |  |  |  |  | -4.6 (-5.6 ,-3.6)* |
| Hungary | 2000-2007 | -1.1 (-4.9 ,2.9) | 2007-2010 | -17.5 (-42.8 ,19.1) | 2010-2018 | -2.4 (-7.4 ,2.9) |  |  | -4.6 (-10.1 ,1.3) |
| Ireland | 2000-2018 | -8.4 (-10.3 ,-6.4)* |  |  |  |  |  |  | -8.4 (-10.3 ,-6.4)* |
| Italy | 2000-2007 | -2.6 (-5.6 ,0.5) | 2007-2014 | -10.9 (-14.5 ,-7.1)* | 2014-2018 | 1.9 (-7.3 ,12.0) |  |  | -5.0 (-7.3 ,-2.5)* |
| Netherlands | 2000-2011 | -8.1 (-9.6 ,-6.6)* | 2011-2018 | -0.6 (-4.8 ,3.8) |  |  |  |  | -5.2 (-6.9 ,-3.5)* |
| Poland | 2000-2018 | -4.0 (-4.6 ,-3.4)* |  |  |  |  |  |  | -4.0 (-4.6 ,-3.4)* |
| Portugal | 2000-2002 | 23.3 (-8.5 ,66.1) | 2002-2012 | -14.1 (-17.2 ,-10.9)* | 2012-2018 | 1.7 (-8.3 ,12.7) |  |  | -5.4 (-9.7 ,-0.9)* |
| Romania | 2000-2005 | -6.0 (-8.1 ,-4.0)* | 2005-2008 | 2.1 (-8.5 ,13.9) | 2008-2011 | -10.1 (-20.6 ,1.9) | 2011-2018 | -4.6 (-6.6 ,-2.7)* | -4.9 (-7.2 ,-2.5)* |
| Slovakia | 2000-2018 | -4.2 (-5.3 ,-3.0)* |  |  |  |  |  |  | -4.2 (-5.3 ,-3.0)* |
| Spain | 2000-2007 | -5.3 (-7.6 ,-2.9)* | 2007-2012 | -19.1 (-25.6 ,-12.1)* | 2012-2018 | -1.5 (-7.2 ,4.6) |  |  | -8.2 (-10.7 ,-5.5)* |
| Sweden | 2000-2018 | -6.1 (-8.6 ,-3.6)* |  |  |  |  |  |  | -6.1 (-8.6 ,-3.6)* |
| Switzerland | 2000-2018 | -5.5 (-6.7 ,-4.4)* |  |  |  |  |  |  | -5.5 (-6.7 ,-4.4)* |
| UK | 2000-2007 | -2.2 (-3.8 ,-0.5)* | 2007-2013 | -11.4 (-14.5 ,-8.2)* | 2013-2018 | 0.3 (-3.9 ,4.8) |  |  | -4.7 (-6.2 ,-3.1)* |
| **EU** | **2001-2008** | **-5.3 (-6.2 ,-4.3)*** | **2008-2012** | **-10.0 (-14.1 ,-5.6)*** | **2012-2017** | **-3.5 (-5.9 ,-1.0)*** |  |  | **-5.9 (-7.1 ,-4.7)*** |
| **Transport accidents** | | | | | | | | | |
| Austria | 2000-2018 | -7.1 (-8.1 ,-6.2)* |  |  |  |  |  |  | -7.1 (-8.1 ,-6.2)* |
| Belgium | 2003-2018 | -8.1 (-9.7 ,-6.6)* |  |  |  |  |  |  | -8.1 (-9.7 ,-6.6)* |
| Bulgaria | 2000-2005 | -3.4 (-8.3 ,1.9) | 2005-2008 | 21.1 (-3.2 ,51.5) | 2008-2011 | -21.6 (-38.8 ,0.3) | 2011-2018 | 2.9 (-1.4 ,7.3) | -0.7 (-5.6 ,4.5) |
| Czechia | 2000-2018 | -5.0 (-6.0 ,-4.1)* |  |  |  |  |  |  | -5.0 (-6.0 ,-4.1)* |
| Denmark | 2000-2008 | -5.1 (-7.7 ,-2.4)* | 2008-2016 | -15.3 (-19.9 ,-10.3)* | 2016-2018 | 35.1 (-13.3 ,110.7) |  |  | -6.1 (-10.7 ,-1.3)* |
| Finland | 2000-2009 | -1.2 (-3.9 ,1.6) | 2009-2012 | -21.1 (-45.4 ,14.1) | 2012-2018 | 6.6 (-0.7 ,14.4) |  |  | -2.4 (-8.1 ,3.6) |
| France | 2001-2017 | -8.2 (-8.8 ,-7.5)* |  |  |  |  |  |  | -8.2 (-8.8 ,-7.5)* |
| Germany | 2000-2018 | -7.7 (-8.3 ,-7.2)* |  |  |  |  |  |  | -7.7 (-8.3 ,-7.2)* |
| Greece | 2000-2009 | -3.4 (-4.7 ,-2.2)* | 2009-2012 | -16.3 (-30.2 ,0.4) | 2012-2015 | 19.8 (-2.8 ,47.7) | 2015-2018 | -19.3 (-27.2 ,-10.5)* | -5.1 (-9.1 ,-1.0)* |
| Hungary | 2000-2007 | 1.5 (-3.4 ,6.6) | 2007-2011 | -19.5 (-36.2 ,1.5) | 2011-2018 | -2.9 (-11.4 ,6.3) |  |  | -5.3 (-10.6 ,0.4) |
| Ireland | 2000-2018 | -9.5 (-11.6 ,-7.4)* |  |  |  |  |  |  | -9.5 (-11.6 ,-7.4)* |
| Italy | 2000-2007 | -3.1 (-5.6 ,-0.5)* | 2007-2014 | -12.4 (-15.6 ,-9.1)* | 2014-2018 | 1.1 (-7.1 ,10.0) |  |  | -5.9 (-8.0 ,-3.8)* |
| Netherlands | 2000-2010 | -10.1 (-12.6 ,-7.4)* | 2010-2018 | -2.5 (-8.1 ,3.4) |  |  |  |  | -6.8 (-9.4 ,-4.2)* |
| Poland | 2000-2018 | -3.4 (-4.1 ,-2.6)* |  |  |  |  |  |  | -3.4 (-4.1 ,-2.6)* |
| Portugal | 2000-2002 | 16.8 (-20.9 ,72.6) | 2002-2018 | -12.0 (-14.1 ,-9.8)* |  |  |  |  | -9.1 (-13.1 ,-5.0)* |
| Romania | 2000-2005 | -5.3 (-11.7 ,1.5) | 2005-2008 | 12.5 (-18.6 ,55.4) | 2008-2013 | -9.9 (-19.5 ,0.9) | 2013-2018 | 1.6 (-7.6 ,11.6) | -2.0 (-7.7 ,4.0) |
| Slovakia | 2000-2018 | -5.1 (-6.7 ,-3.5)* |  |  |  |  |  |  | -5.1 (-6.7 ,-3.5)* |
| Spain | 2000-2007 | -6.5 (-8.7 ,-4.1)* | 2007-2014 | -21.4 (-25.7 ,-16.9)* | 2014-2018 | 10.8 (-4.0 ,27.9) |  |  | -9.2 (-12.4 ,-6.0)* |
| Sweden | 2000-2018 | -8.0 (-9.2 ,-6.7)* |  |  |  |  |  |  | -8.0 (-9.2 ,-6.7)* |
| Switzerland | 2000-2018 | -6.9 (-8.6 ,-5.1)* |  |  |  |  |  |  | -6.9 (-8.6 ,-5.1)* |
| UK | 2000-2007 | -1.3 (-3.2 ,0.7) | 2007-2013 | -19.3 (-22.9 ,-15.6)* | 2013-2018 | -5.2 (-11.6 ,1.7) |  |  | -8.7 (-10.8 ,-6.6)* |
| **EU** | **2001-2008** | **-5.4 (-6.4 ,-4.4)*** | **2008-2013** | **-11.5 (-14.6 ,-8.3)*** | **2013-2017** | **-2.8 (-6.9 ,1.5)** |  |  | **-6.7 (-8.0 ,-5.4)*** |
| **Intentional self-harm** | | | | | | | | | |
| Austria | 2000-2018 | -4.1 (-5.6 ,-2.5)* |  |  |  |  |  |  | -4.1 (-5.6 ,-2.5)* |
| Belgium | 2003-2018 | -2.2 (-4.0 ,-0.3)* |  |  |  |  |  |  | -2.2 (-4.0 ,-0.3)* |
| Bulgaria | 2000-2017 | -0.7 (-3.3 ,2.0) |  |  |  |  |  |  | -0.7 (-3.3 ,2.0) |
| Czechia | 2000-2018 | 0.7 (-0.9 ,2.4) |  |  |  |  |  |  | 0.7 (-0.9 ,2.4) |
| Denmark | 2000-2018 | -1.6 (-3.6 ,0.4) |  |  |  |  |  |  | -1.6 (-3.6 ,0.4) |
| Finland | 2000-2018 | -1.9 (-3.8 ,0.0) |  |  |  |  |  |  | -1.9 (-3.8 ,0.0) |
| France | 2001-2007 | -4.2 (-7.3 ,-0.9)* | 2007-2010 | 5.9 (-13.1 ,29.1) | 2010-2017 | -8.5 (-11.3 ,-5.5)* |  |  | -4.3 (-7.7 ,-0.8)* |
| Germany | 2000-2006 | -6.3 (-10.2 ,-2.3)* | 2006-2018 | -0.6 (-2.3 ,1.1) |  |  |  |  | -2.5 (-4.1 ,-0.9)* |
| Greece | 2000-2002 | -16.9 (NA, NA)* |  |  |  |  |  |  | -16.9 (NA, NA) |
| Hungary | 2000-2018 | -2.6 (-3.8 ,-1.5)* |  |  |  |  |  |  | -2.6 (-3.8 ,-1.5)* |
| Ireland | 2000-2010 | -0.7 (-4.0 ,2.6) | 2010-2018 | -10.9 (-16.5 ,-5.0)* |  |  |  |  | -5.4 (-8.3 ,-2.4)* |
| Italy | 2000-2018 | -0.4 (-1.4 ,0.6) |  |  |  |  |  |  | -0.4 (-1.4 ,0.6) |
| Netherlands | 2000-2018 | 0.6 (-1.2 ,2.4) |  |  |  |  |  |  | 0.6 (-1.2 ,2.4) |
| Poland | 2000-2014 | -0.3 (-1.3 ,0.6) | 2014-2018 | -13.3 (-21.2 ,-4.6)* |  |  |  |  | -3.4 (-5.3 ,-1.4)* |
| Portugal | 2000-2017 | 1.1 (-1.0 ,3.3) |  |  |  |  |  |  | 1.1 (-1.0 ,3.3) |
| Romania | 2000-2018 | -0.8 (-2.4 ,0.9) |  |  |  |  |  |  | -0.8 (-2.4 ,0.9) |
| Slovakia | 2000-2018 | -0.6 (-2.0 ,0.7) |  |  |  |  |  |  | -0.6 (-2.0 ,0.7) |
| Spain | 2000-2018 | -0.8 (-2.6 ,1.1) |  |  |  |  |  |  | -0.8 (-2.6 ,1.1) |
| Sweden | 2000-2018 | 0.3 (-1.2 ,1.8) |  |  |  |  |  |  | 0.3 (-1.2 ,1.8) |
| Switzerland | 2000-2018 | -0.3 (-2.2 ,1.7) |  |  |  |  |  |  | -0.3 (-2.2 ,1.7) |
| UK | 2000-2003 | -14.1 (-22.6 ,-4.8)* | 2003-2014 | 0.7 (-1.0 ,2.5) | 2014-2018 | 11.3 (4.7 ,18.4)* |  |  | 0.3 (-1.9 ,2.5) |
| **EU** | **2001-2017** | **-2.1 (-2.5 ,-1.7)*** |  |  |  |  |  |  | **-2.1 (-2.5 ,-1.7)*** |
| **Poisoning** | | | | | | | | | |
| Bulgaria | 2000-2004 | -13.2 (-73.1 ,179.9) |  |  |  |  |  |  | -13.2 (-73.1 ,179.9) |
| Finland | 2003-2018 | 1.4 (-3.7 ,6.8) |  |  |  |  |  |  | 1.4 (-3.7 ,6.8) |
| France | 2001-2016 | -1.6 (-5.7 ,2.6) |  |  |  |  |  |  | -1.6 (-5.7 ,2.6) |
| Germany | 2000-2005 | -14.8 (-24.8 ,-3.5)* | 2005-2018 | 2.0 (-1.9 ,6.0) |  |  |  |  | -3.0 (-6.7 ,0.8) |
| Greece | 2000-2010 | -10.3 (-20.1 ,0.7) |  |  |  |  |  |  | -10.3 (-20.1 ,0.7) |
| Poland | 2000-2018 | -2.1 (-5.2 ,1.1) |  |  |  |  |  |  | -2.1 (-5.2 ,1.1) |
| Romania | 2000-2018 | -9.5 (-11.2 ,-7.7)* |  |  |  |  |  |  | -9.5 (-11.2 ,-7.7)* |
| Spain | 2000-2008 | -8.1 (-12.9 ,-3.1)* |  |  |  |  |  |  | -8.1 (-12.9 ,-3.1)* |
| Sweden | 2011-2018 | 1.7 (-5.1 ,8.9) |  |  |  |  |  |  | 1.7 (-5.1 ,8.9) |
| UK | 2000-2004 | -12.9 (-27.6 ,4.8) | 2004-2018 | 4.2 (1.4 ,7.2)* |  |  |  |  | 0.2 (-4.0 ,4.5) |
| **EU** | **2001-2012** | **-7.6 (-9.1 ,-6.0)*** | **2012-2017** | **1.5 (-5.4 ,8.9)** |  |  |  |  | **-4.8 (-6.9 ,-2.7)*** |
| **Assault** | | | | | | | | | |
| Belgium | 2003-2018 | -3.1 (-7.5 ,1.6) |  |  |  |  |  |  | -3.1 (-7.5 ,1.6) |
| Bulgaria | 2000-2004 | -3.7 (-27.7 ,28.2) |  |  |  |  |  |  | -3.7 (-27.7 ,28.2) |
| Czechia | 2001-2007 | 4.0 (NA, NA)* |  |  |  |  |  |  | 4.0 (NA, NA) |
| France | 2001-2017 | -3.9 (-5.5 ,-2.3)* |  |  |  |  |  |  | -3.9 (-5.5 ,-2.3)* |
| Germany | 2000-2018 | -2.1 (-4.0 ,-0.1)* |  |  |  |  |  |  | -2.1 (-4.0 ,-0.1)* |
| Hungary | 2000-2018 | -0.1 (-2.2 ,2.1) |  |  |  |  |  |  | -0.1 (-2.2 ,2.1) |
| Italy | 2000-2018 | -3.0 (-4.7 ,-1.3)* |  |  |  |  |  |  | -3.0 (-4.7 ,-1.3)* |
| Netherlands | 2000-2013 | -1.9 (-5.6 ,1.9) |  |  |  |  |  |  | -1.9 (-5.6 ,1.9) |
| Poland | 2000-2014 | -5.0 (-7.3 ,-2.7)* |  |  |  |  |  |  | -5.0 (-7.3 ,-2.7)* |
| Romania | 2000-2016 | -6.7 (-8.6 ,-4.7)* |  |  |  |  |  |  | -6.7 (-8.6 ,-4.7)* |
| Spain | 2001-2018 | -2.6 (-5.0 ,-0.1)* |  |  |  |  |  |  | -2.6 (-5.0 ,-0.1)* |
| UK | 2000-2002 | -30.2 (-48.9 ,-4.5)* | 2002-2014 | -6.3 (-9.4 ,-3.0)* |  |  |  |  | -10.1 (-14.1 ,-5.9)* |
| **EU** | **2001-2017** | **-5.1 (-5.6 ,-4.6)*** |  |  |  |  |  |  | **-5.1 (-5.6 ,-4.6)*** |

Supplementary Table 1: girls

| **Country** | **Trend 1** | **APC 1** | **Trend 2** | **APC 2** | **Trend 3** | **APC 3** | **AAPC** |
| --- | --- | --- | --- | --- | --- | --- | --- |
| **External causes** | | | | | | | |
| Austria | 2000-2018 | -5.1 (-6.7 ,-3.6)* |  |  |  |  | -5.1 (-6.7 ,-3.6)* |
| Belgium | 2003-2018 | -5.0 (-6.3 ,-3.6)* |  |  |  |  | -5.0 (-6.3 ,-3.6)* |
| Bulgaria | 2000-2018 | -4.7 (-5.9 ,-3.6)* |  |  |  |  | -4.7 (-5.9 ,-3.6)* |
| Czechia | 2000-2018 | -4.3 (-5.3 ,-3.2)* |  |  |  |  | -4.3 (-5.3 ,-3.2)* |
| Denmark | 2000-2018 | -6.2 (-7.6 ,-4.7)* |  |  |  |  | -6.2 (-7.6 ,-4.7)* |
| Finland | 2000-2018 | -3.4 (-5.3 ,-1.5)* |  |  |  |  | -3.4 (-5.3 ,-1.5)* |
| France | 2001-2004 | -13.6 (-17.8 ,-9.3)* | 2004-2017 | -3.3 (-3.9 ,-2.6)* |  |  | -5.3 (-6.2 ,-4.4)* |
| Germany | 2000-2006 | -7.0 (-9.1 ,-4.8)* | 2006-2018 | -3.8 (-4.9 ,-2.8)* |  |  | -4.9 (-5.8 ,-4.0)* |
| Greece | 2000-2018 | -3.1 (-5.7 ,-0.4)* |  |  |  |  | -3.1 (-5.7 ,-0.4)* |
| Hungary | 2000-2018 | -5.0 (-5.9 ,-4.1)* |  |  |  |  | -5.0 (-5.9 ,-4.1)* |
| Ireland | 2000-2018 | -7.4 (-9.2 ,-5.6)* |  |  |  |  | -7.4 (-9.2 ,-5.6)* |
| Italy | 2000-2018 | -5.4 (-6.4 ,-4.4)* |  |  |  |  | -5.4 (-6.4 ,-4.4)* |
| Netherlands | 2000-2018 | -3.7 (-4.7 ,-2.7)* |  |  |  |  | -3.7 (-4.7 ,-2.7)* |
| Poland | 2000-2018 | -3.6 (-4.4 ,-2.8)* |  |  |  |  | -3.6 (-4.4 ,-2.8)* |
| Portugal | 2000-2018 | -8.3 (-9.8 ,-6.8)* |  |  |  |  | -8.3 (-9.8 ,-6.8)* |
| Romania | 2000-2018 | -5.2 (-5.7 ,-4.6)* |  |  |  |  | -5.2 (-5.7 ,-4.6)* |
| Slovakia | 2000-2018 | -3.5 (-4.8 ,-2.1)* |  |  |  |  | -3.5 (-4.8 ,-2.1)* |
| Spain | 2000-2012 | -8.0 (-9.6 ,-6.4)* | 2012-2018 | -2.2 (-8.6 ,4.7) |  |  | -6.1 (-8.3 ,-3.9)* |
| Sweden | 2000-2018 | -3.7 (-6.4 ,-0.8)* |  |  |  |  | -3.7 (-6.4 ,-0.8)* |
| Switzerland | 2000-2018 | -3.8 (-5.2 ,-2.3)* |  |  |  |  | -3.8 (-5.2 ,-2.3)* |
| UK | 2000-2008 | -2.1 (-3.8 ,-0.3)* | 2008-2012 | -15.2 (-23.4 ,-6.2)* | 2012-2018 | 3.7 (-0.1 ,7.7) | -3.3 (-5.6 ,-1.0)* |
| **EU** | **2001-2008** | **-4.9 (-5.6 ,-4.2)*** | **2008-2012** | **-7.8 (-10.9 ,-4.5)*** | **2012-2017** | **-2.4 (-4.2 ,-0.7)*** | **-4.9 (-5.8 ,-4.0)*** |
| **Accidents** | | | | | | | |
| Austria | 2000-2018 | -7.0 (-8.8 ,-5.2)* |  |  |  |  | -7.0 (-8.8 ,-5.2)* |
| Belgium | 2003-2018 | -5.9 (-8.0 ,-3.7)* |  |  |  |  | -5.9 (-8.0 ,-3.7)* |
| Bulgaria | 2000-2018 | -5.1 (-6.4 ,-3.7)* |  |  |  |  | -5.1 (-6.4 ,-3.7)* |
| Czechia | 2000-2018 | -6.0 (-7.4 ,-4.7)* |  |  |  |  | -6.0 (-7.4 ,-4.7)* |
| Denmark | 2000-2016 | -7.2 (-9.4 ,-4.8)* |  |  |  |  | -7.2 (-9.4 ,-4.8)* |
| Finland | 2000-2018 | -5.3 (-7.7 ,-2.8)* |  |  |  |  | -5.3 (-7.7 ,-2.8)* |
| France | 2001-2004 | -14.7 (-21.5 ,-7.4)* | 2004-2017 | -4.0 (-5.1 ,-2.9)* |  |  | -6.1 (-7.6 ,-4.6)* |
| Germany | 2000-2018 | -6.7 (-7.3 ,-6.0)* |  |  |  |  | -6.7 (-7.3 ,-6.0)* |
| Greece | 2000-2018 | -3.4 (-6.3 ,-0.4)* |  |  |  |  | -3.4 (-6.3 ,-0.4)* |
| Hungary | 2000-2018 | -6.2 (-7.3 ,-5.0)* |  |  |  |  | -6.2 (-7.3 ,-5.0)* |
| Ireland | 2000-2011 | -8.0 (-10.7 ,-5.2)* |  |  |  |  | -8.0 (-10.7 ,-5.2)* |
| Italy | 2000-2018 | -6.3 (-7.4 ,-5.1)* |  |  |  |  | -6.3 (-7.4 ,-5.1)* |
| Netherlands | 2000-2018 | -6.3 (-7.7 ,-4.9)* |  |  |  |  | -6.3 (-7.7 ,-4.9)* |
| Poland | 2000-2018 | -4.3 (-5.2 ,-3.4)* |  |  |  |  | -4.3 (-5.2 ,-3.4)* |
| Portugal | 2000-2003 | 13.3 (-8.3 ,40.0) | 2003-2006 | -26.0 (-54.9 ,21.5) | 2006-2018 | -6.0 (-10.0 ,-1.9)* | -6.8 (-14.3 ,1.3) |
| Romania | 2000-2018 | -5.7 (-6.3 ,-5.0)* |  |  |  |  | -5.7 (-6.3 ,-5.0)* |
| Slovakia | 2000-2018 | -4.1 (-5.4 ,-2.8)* |  |  |  |  | -4.1 (-5.4 ,-2.8)* |
| Spain | 2000-2018 | -8.4 (-9.4 ,-7.3)* |  |  |  |  | -8.4 (-9.4 ,-7.3)* |
| Sweden | 2000-2018 | -7.0 (-11.7 ,-2.0)* |  |  |  |  | -7.0 (-11.7 ,-2.0)* |
| Switzerland | 2000-2018 | -3.2 (-5.4 ,-1.0)* |  |  |  |  | -3.2 (-5.4 ,-1.0)* |
| EU | 2001-2008 | -5.6 (-6.4 ,-4.8)* | 2008-2013 | -8.8 (-11.2 ,-6.4)* | 2013-2017 | -3.0 (-5.9 ,0.1) | -6.0 (-7.0 ,-5.0)* |
| **UK** | **2000-2008** | **-1.4 (-3.6 ,0.9)** | **2008-2011** | **-17.5 (-35.0 ,4.7)** | **2011-2018** | **-1.0 (-4.7 ,2.9)** | **-4.1 (-7.8 ,-0.3)*** |
| **Transport accidents** | | | | | | | |
| Austria | 2000-2017 | -5.9 (-8.4 ,-3.4)* |  |  |  |  | -5.9 (-8.4 ,-3.4)* |
| Belgium | 2003-2018 | -6.9 (-9.3 ,-4.5)* |  |  |  |  | -6.9 (-9.3 ,-4.5)* |
| Bulgaria | 2000-2018 | -3.8 (-6.4 ,-1.0)* |  |  |  |  | -3.8 (-6.4 ,-1.0)* |
| Czechia | 2000-2018 | -6.5 (-8.4 ,-4.5)* |  |  |  |  | -6.5 (-8.4 ,-4.5)* |
| Denmark | 2000-2016 | -5.3 (-7.6 ,-3.0)* |  |  |  |  | -5.3 (-7.6 ,-3.0)* |
| Finland | 2000-2015 | -3.7 (-6.2 ,-1.2)* |  |  |  |  | -3.7 (-6.2 ,-1.2)* |
| France | 2001-2003 | -23.8 (-38.9 ,-5.1)* | 2003-2017 | -6.0 (-7.5 ,-4.5)* |  |  | -8.5 (-11.0 ,-5.9)* |
| Germany | 2000-2018 | -7.9 (-8.6 ,-7.2)* |  |  |  |  | -7.9 (-8.6 ,-7.2)* |
| Greece | 2000-2012 | -7.0 (-10.2 ,-3.6)* | 2012-2015 | 41.9 (-39.9 ,235.4) | 2015-2018 | -42.7 (-62.0 ,-13.8)* | -7.9 (-20.2 ,6.2) |
| Hungary | 2000-2004 | 3.6 (-7.1 ,15.6) | 2004-2018 | -7.8 (-10.0 ,-5.5)* |  |  | -5.4 (-8.0 ,-2.7)* |
| Ireland | 2000-2011 | -7.7 (-11.0 ,-4.2)* |  |  |  |  | -7.7 (-11.0 ,-4.2)* |
| Italy | 2000-2006 | -2.5 (-9.4 ,5.0) | 2006-2018 | -9.8 (-11.6 ,-8.0)* |  |  | -7.4 (-9.7 ,-5.1)* |
| Netherlands | 2000-2018 | -7.4 (-9.0 ,-5.7)* |  |  |  |  | -7.4 (-9.0 ,-5.7)* |
| Poland | 2000-2018 | -3.9 (-4.8 ,-2.9)* |  |  |  |  | -3.9 (-4.8 ,-2.9)* |
| Portugal | 2000-2018 | -9.6 (-12.8 ,-6.3)* |  |  |  |  | -9.6 (-12.8 ,-6.3)* |
| Romania | 2000-2018 | -2.9 (-4.2 ,-1.6)* |  |  |  |  | -2.9 (-4.2 ,-1.6)* |
| Slovakia | 2000-2018 | -3.9 (-5.9 ,-1.9)* |  |  |  |  | -3.9 (-5.9 ,-1.9)* |
| Spain | 2000-2018 | -10.3 (-11.7 ,-8.8)* |  |  |  |  | -10.3 (-11.7 ,-8.8)* |
| Sweden | 2000-2018 | -4.6 (-7.5 ,-1.6)* |  |  |  |  | -4.6 (-7.5 ,-1.6)* |
| Switzerland | 2000-2016 | -2.2 (-5.0 ,0.8) |  |  |  |  | -2.2 (-5.0 ,0.8) |
| UK | 2000-2008 | 0.3 (-2.1 ,2.9) | 2008-2011 | -30.2 (-47.9 ,-6.3)* | 2011-2018 | -5.2 (-10.6 ,0.5) | -7.6 (-12.0 ,-2.9)* |
| **EU** | **2001-2008** | **-5.8 (-7.3 ,-4.2)*** | **2008-2011** | **-12.5 (-25.0 ,2.2)** | **2011-2017** | **-5.3 (-8.3 ,-2.2)*** | **-6.9 (-9.5 ,-4.2)*** |
|  |  |  |  |  |  |  |  |
| **Intentional self-harm** | | | | | | | |
| Austria | 2000-2016 | -1.5 (-4.4 ,1.6) |  |  |  |  | -1.5 (-4.4 ,1.6) |
| Belgium | 2003-2018 | -3.0 (-5.0 ,-1.0)* |  |  |  |  | -3.0 (-5.0 ,-1.0)* |
| Bulgaria | 2001-2007 | -0.1 (-16.9 ,20.0) |  |  |  |  | -0.1 (-16.9 ,20.0) |
| Czechia | 2000-2018 | 2.6 (0.1 ,5.2)* |  |  |  |  | 2.6 (0.1 ,5.2)* |
| Finland | 2000-2018 | -0.4 (-2.2 ,1.5) |  |  |  |  | -0.4 (-2.2 ,1.5) |
| France | 2001-2007 | -4.0 (-7.6 ,-0.2)* | 2007-2010 | 8.3 (-14.0 ,36.5) | 2010-2017 | -3.5 (-6.5 ,-0.5)* | -1.6 (-5.5 ,2.6) |
| Germany | 2000-2008 | -3.6 (-6.0 ,-1.1)* | 2008-2016 | 6.4 (3.2 ,9.8)* | 2016-2018 | -15.4 (-33.6 ,7.7) | -0.7 (-3.5 ,2.2) |
| Hungary | 2000-2017 | 0.7 (-0.9 ,2.3) |  |  |  |  | 0.7 (-0.9 ,2.3) |
| Ireland | 2006-2016 | 2.6 (-26.2 ,42.7) |  |  |  |  | 2.6 (-26.2 ,42.7) |
| Italy | 2000-2018 | 1.1 (-1.3 ,3.6) |  |  |  |  | 1.1 (-1.3 ,3.6) |
| Netherlands | 2001-2018 | 4.8 (2.6 ,6.9)* |  |  |  |  | 4.8 (2.6 ,6.9)* |
| Poland | 2000-2018 | 1.2 (-0.4 ,2.9) |  |  |  |  | 1.2 (-0.4 ,2.9) |
| Romania | 2000-2018 | -0.0 (-2.0 ,2.1) |  |  |  |  | -0.0 (-2.0 ,2.1) |
| Spain | 2000-2011 | -2.8 (-6.4 ,0.9) | 2011-2014 | 25.3 (-14.5 ,83.7) | 2014-2018 | -5.0 (-15.1 ,6.4) | 0.9 (-5.2 ,7.4) |
| Sweden | 2000-2018 | 1.4 (-0.6 ,3.4) |  |  |  |  | 1.4 (-0.6 ,3.4) |
| Switzerland | 2001-2017 | -1.9 (-3.8 ,-0.0)* |  |  |  |  | -1.9 (-3.8 ,-0.0)* |
| UK | 2000-2013 | -1.6 (-4.2 ,1.0) | 2013-2018 | 19.0 (8.8 ,30.2)* |  |  | 3.7 (0.8 ,6.7)* |
| **EU** | **2001-2017** | **0.6 (-0.0 ,1.3)** |  |  |  |  | **0.6 (-0.0 ,1.3)** |
| **Poisoning** | | | | | | | |
| France | 2001-2007 | 8.1 (-5.3 ,23.2) |  |  |  |  | 8.1 (-5.3 ,23.2) |
| Germany | 2000-2018 | 1.1 (0.4 ,1.8)* |  |  |  |  | 1.1 (0.4 ,1.8)* |
| Poland | 2000-2017 | -2.3 (-5.1 ,0.5) |  |  |  |  | -2.3 (-5.1 ,0.5) |
| Romania | 2000-2018 | -8.5 (-10.1 ,-7.0)* |  |  |  |  | -8.5 (-10.1 ,-7.0)* |
| Spain | 2000-2001 | 17.5 (NA, NA)* |  |  |  |  | 17.5 (NA, NA) |
| UK | 2000-2018 | 0.1 (-1.7 ,2.0) |  |  |  |  | 0.1 (-1.7 ,2.0) |
| **EU** | **2001-2017** | **-6.0 (-7.1 ,-4.9)*** |  |  |  |  | **-6.0 (-7.1 ,-4.9)*** |
| **Assault** | | | | | | | |
| Belgium | 2003-2006 | -2.5 (-31.0 ,37.9) |  |  |  |  | -2.5 (-31.0 ,37.9) |
| Finland | 2002-2012 | 0.9 (NA, NA)* |  |  |  |  | 0.9 (NA, NA) |
| France | 2001-2017 | -4.2 (-6.9 ,-1.5)* |  |  |  |  | -4.2 (-6.9 ,-1.5)* |
| Germany | 2000-2018 | -2.3 (-4.3 ,-0.2)* |  |  |  |  | -2.3 (-4.3 ,-0.2)* |
| Hungary | 2002-2007 | -1.4 (-23.0 ,26.4) |  |  |  |  | -1.4 (-23.0 ,26.4) |
| Italy | 2000-2014 | -1.3 (-4.7 ,2.3) |  |  |  |  | -1.3 (-4.7 ,2.3) |
| Netherlands | 2000-2006 | 1.8 (-4.0 ,7.9) |  |  |  |  | 1.8 (-4.0 ,7.9) |
| Poland | 2000-2012 | -4.7 (-8.3 ,-0.9)* |  |  |  |  | -4.7 (-8.3 ,-0.9)* |
| Romania | 2000-2018 | -3.8 (-6.0 ,-1.5)* |  |  |  |  | -3.8 (-6.0 ,-1.5)* |
| Spain | 2004-2018 | -5.7 (-9.4 ,-1.8)* |  |  |  |  | -5.7 (-9.4 ,-1.8)* |
| UK | 2000-2002 | -38.5 (-62.3 ,0.3) | 2002-2009 | -1.7 (-9.2 ,6.5) |  |  | -11.4 (-19.5 ,-2.6)* |
| **EU** | **2001-2017** | **-4.6 (-5.9 ,-3.3)*** |  |  |  |  | **-4.6 (-5.9 ,-3.3)*** |
